# In‐frame deletion of SMC5 related with the phenotype of primordial dwarfism, chromosomal instability and insulin resistance

**DOI:** 10.1002/ctm2.1007

**Published:** 2023-01-10

**Authors:** Wenjiao Zhu, Yuanping Shi, Changrun Zhang, Yajie Peng, Yueyue Wan, Yue Xu, Xuemeng Liu, Bing Han, Shuangxia Zhao, Yanping Kuang, Huaidong Song, Jie Qiao

**Affiliations:** ^1^ Department of Endocrinology Shanghai Ninth People's Hospital Shanghai Jiao Tong University School of Medicine Shanghai China; ^2^ Department of Molecular Diagnostics & Endocrinology The Core Laboratory in Medical Center of Clinical Research Shanghai Ninth People's Hospital State Key Laboratory of Medical Genomics Shanghai Jiao Tong University School of Medicine Shanghai China; ^3^ Department of Assisted Reproduction Shanghai Ninth People's Hospital Shanghai Jiao Tong University School of Medicine Shanghai China

**Keywords:** insulin resistance, primordial dwarfism, SMC5 mutation

## Abstract

**Background:**

SMC5/6 complex plays a vital role in maintaining genome stability, yet the relationship with human diseases has not been described.

**Methods:**

SMC5 variation was identified through whole‐exome sequencing (WES) and verified by Sanger sequencing. Immunoprecipitation, cytogenetic analysis, fluorescence activated cell sorting (FACS) and electron microscopy were used to elucidate the cellular consequences of patient's cells. smc5 knockout (KO) zebrafish and Smc5^K371del^ knock‐in mouse models were generated by CRISPR‐Cas9. RNA‐seq, quantitative real‐time PCR (qPCR), western blot, microquantitative computed tomography (microCT) and histology were used to explore phenotypic characteristics and potential mechanisms of the animal models. The effects of Smc5 knockdown on mitotic clonal expansion (MCE) during adipogenesis were investigated through Oil Red O staining, proliferation and apoptosis assays in vitro.

**Results:**

We identified a homozygous in‐frame deletion of Arg372 in *SMC5*, one of the core subunits of the SMC5/6 complex, from an adult patient with microcephalic primordial dwarfism, chromosomal instability and insulin resistance. SMC5 mutation disrupted its interaction with its interacting protein NSMCE2, leading to defects in DNA repair and chromosomal instability in patient fibroblasts. Smc5 KO zebrafish showed microcephaly, short length and disturbed glucose metabolism. Smc5 depletion triggers a p53‐related apoptosis, as concomitant deletion of the p53 rescued growth defects phenotype in zebrafish. An smc5^K371del^ knock‐in mouse model exhibited high mortality, severe growth restriction and fat loss. In 3T3‐L1 cells, the knockdown of smc5 results in impaired MCE, a crucial step in adipogenesis. This finding implies that defective cell survival and differentiation is an important mechanism linking growth disorders and metabolic homeostasis imbalance.

## Graphical abstract

In‐frame Arg372 deletion of SMC5 gene causes dwarfism, diabetes and extreme insulin resistance. SMC5 depletion generates DNA damage that activates p53‐related pathway. Both zebrafish KO and mouse KI models show poor survival, short stature and glucose intolerance. SMC5 deficiency impairs adipocyte proliferation and differentiation.

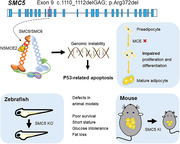

## 1 INTRODUCTION

The SMC5/6 complex, along with the cohesin and condensin complexes, belongs to the structural maintenance of chromosomes (SMC) family of protein complexes, which play important roles in chromosome architecture and dynamics. Unlike cohesin and condensin, which have well‐established functions in chromosome structure, the SMC5/6 complex lacks functional nomenclature, although some evidence suggests important roles in chromosome segregation and DNA repair.^1^ Recently, the SMC5/6 complex was found to act as an ATP‐dependent intermolecular DNA linker by modulating the remodelling of recombination intermediates caused by endogenous replication stress, and mediating replication via natural pausing sites.^2^, ^3^ To date, two genomic instability syndromes with totally different clinical features have been reported due to mutations in non‐SMC elements of SMC5/6^4,5^. However, the core elements (SMC5 and SMC6) mutation has not been disclosed to be associated with human phenotype. Owing to the embryonic lethality in the knockout (KO) mice, most studies of this enigmatic complex were performed in the *Saccharomyces cerevisiae* and depleted cells.^6^, ^7^

Microcephalic primordial dwarfism (MPD) is a group of rare monogenic disorders that are characterized by extreme reductions in brain and body size from an early developmental stage. MPD comprises several phenotypically distinct Mendelian disorders, such as Seckel syndrome and microcephalic osteodysplastic primordial dwarfism Types 1 and 2.^8^, ^9^ Interestingly, in a number of complex syndromes, MPD occurs in combination with insulin resistance status, which is a complex pathological state of impaired cellular response to insulin in target cells such as adipocytes. Pathogenic genes have been identified to be involved in centrosome dysfunction, impaired DNA damage repair or genomic instability.^10^ Although there is distinct evidence that insulin resistance occurs in syndromes featuring dwarfism, the potential link between insulin sensitivity and cell autonomous defects in DNA replication and/or DNA damage responses remains unclear.

In this study, we identified a homozygous in‐frame mutation in core element *SMC5*, resulting in primordial dwarfism and insulin‐resistant diabetes. The phenotype is further supported by results from both smc5 KO zebrafish and knock‐in (KI) mouse models. The activation of P53 signal pathway and the consequent apoptosis induced by *SMC5* deficiency could be responsible for the primordial dwarfism, and the disrupted mitotic clonal expansion during adipogenesis underlies the phenotypes of insulin resistance.

## 2 METHODS AND MATERIALS

### 2.1 Whole‐exome sequencing (WES) and variant analysis

Genomic DNA from trio samples was isolated and amplified on an Agilent SureSelect Human All Exon Kit, followed by two‐end sequencing (150 bp each) at an Illumina HiSeq 2500 platform as previously described.^11^ The sequence reads were aligned to the human reference genome (GRCh37) with BWA (version .7.7).^12^ Following alignment, reads were recalibrated, base quality scores were recalculated, and indel realignment was performed using GATK 2.0.^13^ Indels and single nucleotide variants were identified with GATK Unified Genotyper^13^ and SAMtools (version .1.19)^14^ and annotated using ANNOVAR.^15^ Candidate autosomal recessive mutational events were identified with a focus on protein alterations and splice site changes, according to the following criteria: (1) high‐quality sequence reads (variant site quality ≥50; mapping quality ≥50) and a minimum of five total reads for the proband; (2) minor allele frequency ≤10^−3^ in the 1000 Genomes Project, Exome Sequencing Project (ESP6500I), Exome Aggregation Consortium (ExAC), dbSNP135 and our internal exome database (175 normal individuals); (3) mutation pathogenicity was assessed using PolyPhen‐2 and SIFT prediction software^16^ and filtered if considered not damaging by either software; and (4) homozygous. Eleven candidate variants remained (Table S3) and were manually investigated for potential novel–inherited disease‐causing genes. Sanger sequencing was conducted to validate mutations in *SMC5* using primers: 5′‐GTCTGGTAGAACTGGTAATGTGC‐3′ and 5′‐ATCTGGGGCTGAAGATTCTCG‐3′. The genome‐wide copy number analysis of the proband was conducted using Affymetrix CytoScan HD arrays (Affymetrix, Santa Clara, CA, US) in accordance with the manufacturer's instructions. Affymetrix Chromosome Analysis Suite was used to analyse the results as described previously.^17^

### 2.2 Plasmids and virus

For Co‐IP assay, the complete open reading frame of human SMC5, SMC6 and NSMCE2 was amplified from HEK293T cell cDNA with PCR using oligonucleotides carrying the Flag, Myc, His epitope tag at the C terminus, respectively, and cloned into the pCDNA3.1 vector using 5′ KpnI and 3′ NotI restriction sites. The R372del mutation of SMC5 was generated using the Fast Mutagenesis System (TransGen Biotech). For rescue experiments in zebrafish, human wild‐type (WT) or R372del mutant SMC5 cDNA was cloned into the Gateway Tol2 vector (pDestTol2pA2) with a green fluorescent protein (GFP) reporter under a constitutive promoter (tol2‐ubiquitin‐hSMC5‐2A‐EGFP) using 5′ SalI and 3′ EcoRV restriction sites. For a knockdown of SMC5, human and mouse SMC5 shRNA target sequences were cloned into pHBLV‐U6‐MCS‐CMV‐ZsGreen PGK PURO vectors (HANBIO Inc.), or human single‐guide RNAs (sgRNAs) targeting SMC5 were cloned into pHBLV‐U6‐gRNA‐EF1‐CAS9‐PURO vectors (HANBIO Inc.). We prepared the lentiviral particles by transiently transfecting 293T cells with lentiviral vectors as per standard protocols.^18^ A scramble control vector was cloned in parallel. The shRNA and sgRNA sequences targeting SMC5 sites are detailed in Table S5.

### 2.3 Cell culture, transfections and transductions

Dermal fibroblasts were obtained from skin biopsies and cultured in fibroblast medium (ScienCell). Cells, such as 293T, 3T3‐L1 and HepG2, were obtained from ATCC. 293T cells, 3T3‐L1 preadipocyte and mouse embryonic fibroblasts (MEFs) isolated from E13.5 embryos were kept in DMEM with 10% fetal bovine serum (FBS). The HepG2 cells were cultured in MEM medium supplemented with 10% FBS.

For immunoprecipitation assay, 293T cells were grown to 70% confluence on a 10‐cm dish, then cotransfected with 5‐μg pcDNA3.1‐Myc‐SMC6 plasmid (Myc‐SMC6) or 5‐μg pcDNA3.1‐His‐NSMCE2 (His‐NSMCE2) and 5‐μg pcDNA3.1‐3xFlag‐SMC5 (3xFlag‐SMC5 WT or R372DEL) using a Lipofectamine 2000 Reagent (Invitrogen).

Differentiation of 3T3‐L1 cells was induced in the medium consisting of DMEM with 10% FBS, .5‐mM 3‐isobutyl‐1‐methylxanthine, 1‐μM dexamethasone and 10‐mg/ml insulin. After 2 days of induction, the medium was replaced by DMEM containing 10% FBS and 10‐mg/ml insulin only and then changed every 2 days with DMEM plus 10% FBS until the cells became mature adipocytes. The differentiation of 3T3‐L1 cells upon Smc5 knockdown was investigated using lentiviruses transduced at an multiplicity of infection (MOI) of 20 4 days prior to induction of differentiation (D‐4, unless stated otherwise). The differentiation of MEFs is similar to that of 3T3‐L1 cells, except that rosiglitazone (2.5 μM, Sigma) is added to the two cocktails. Oil red O staining was implemented to measure triglyceride contents in mature adipocytes as previously described.^19^ Viability of 3T3‐L1 cells was tested using the CellTiter‐Glo assay (Promega) according to the manufacturer's instruction.

The knockdown of SMC5 in HepG2 cells was carried out using lentiviruses transduced with shRNAs or sgRNAs at an MOI of 10.

### 2.4 Electron microscopy

Patient and control fibroblasts were fixed in 2.5% glutaraldehyde overnight (.01 M, pH = 7.4), post‐fixed in 1% osmium tetroxide and then dehydrated in a graded series of ethanol. Finally, the samples were embedded in pure epoxy resin. Ultrathin sections were obtained with an LKB ultramicrotome, stained with lead citrate and observed using a transmission electron microscope (Hitachi, H‐7650, Tokyo, Japan). For each group, at least 100 cells from randomly chosen fields were observed.

### 2.5 Cytogenetic analysis

Seeded fibroblasts were treated with 1‐mM hydroxyurea (HU, H8627, Sigma) or saline for 4 h, recovered in the presence of cytochalasin B (3 μg/ml) for 48 h, followed by staining with Alexa 488 Phalloidin (Cytoskeleton Inc.) and 4,6‐diamidino‐2‐ phenylindole (DAPI, Invitrogen) then mounted on glass slides. Cells were imaged using an Axio Scan.Z1 automatic Digital Slide Scanner (Zeiss, Germany) with × 40 magnification. Approximately 900 binucleated fibroblasts were analysed for micronucleus (MN) and nucleoplasmic bridge (NPB).

The frequency of chromosomal aberrations and MN in lymphocytes were performed under basal condition according to the international standard protocol^20^ with some modifications. For each subject, a total of 100 well‐spread metaphases were analysed. Aberrations, including acentric fragments, dicentrics, rings, pericentric inversions, translocations and interstitial deletions, were recorded, summed and reported. To detect MN, lymphocytes were incubated for 72 h and stained with Giemsa as in chromosomal aberrations assay. For each subject, 1000 cells with well‐preserved cytoplasm were examined, and micronuclei were expressed as the number of micronuclei per 1000 analysed cells.

### 2.6 Fluorescence‐activated cell sorting (FACS)

Cells were stained with propidium iodide (Sigma) for the analysis of cell cycle as previously described.^7^ For apoptosis assay, Annexin V and PI staining was performed on 2 × 10^5^ cells with a Dead Cell Apoptosis Kit (Life Technologies, V13242). The cells were analysed using a CytoFLEX LX Flow Cytometer (Beckman Coulter). The data were collected and analysed with FlowJo and ModFit software.

### 2.7 Western blot and immunoprecipitation

Total cellular proteins were lysed in radioimmunoprecipitation assay buffer containing protease and phosphatase inhibitors. Proteins were separated by SDS–PAGE electrophoresis and transferred to polyvinylidene fluoride membranes (Amersham International). Membranes were blocked in 5% bovine serum albumin for 1 h and then incubated overnight at 4°C using the following antibodies: Flag (ab49763, Abcam; 1:500), 6xHis (ab184607, Abcam; 1:1000), Myc (ab1326, Abcam; 1:1000), SMC5 (NB100‐469, NOVUS; 1:2000), SMC5 (SAB2700100, Sigma; 1:1000), SMC5 (PA5‐30460, Thermo Fisher; 1:1000), SMC6L1 (H00079677‐M01, Abnova; 1:1000), NSMCE2 (ab105363, Abcam; 1:1000), PPARγ (2435, Cell Signaling; 1:1000), C/EBPα (2295, Cell Signaling; 1:1000), GLUT4 (ab33780, Abcam; 1:1000), AKT (9272, Cell Signaling; 1:1000), phospho‐AKT (4051, Cell Signaling; 1:1000), tubulin (ab21058, Abcam; 1:1000) followed by incubation with horseradish peroxidase–conjugated anti‐rabbit (ab205718, Abcam; 1:2000) or anti‐mouse (7076, Cell Signaling; 1:1000) secondary antibodies for 2 h at room temperature. Immunoblots were developed using an enhanced chemiluminescence detection system (Amersham 600, GE).

For immunoprecipitation, cells were collected 48‐h post‐transfection, and FLAG‐tagged proteins were immunoprecipitated with EZview Red ANTI‐FLAG M2 Affinity Gel (F2426, Sigma) according to the manufacturer's instructions. Simply, cells were lysed with a 1‐ml lysis buffer (50‐mM Tris–HCl, pH 7.4, 150‐mM NaCl, 1‐mM EDTA, 1% Triton X‐100, protease inhibitors [Roche]). Cell lysate supernatant was incubated with 40 ‐μl anti‐FLAG resin overnight at 4°C. The beads were washed thrice with a 1‐ ml Wash buffer and then resuspended in SDS‐loading buffer for analyses by immunoblotting.

### 2.8 Zebrafish models

Zebrafish were maintained and staged using standard protocols as previously described.^21^ For knockdown smc5, morpholino (MO) oligonucleotides (Table S6) were designed by Gene Tools, LLC and microinjected into one‐cell‐stage zebrafish embryos as previously described.^21^ A CRISPR‐Cas9 system was used to produce the smc5 KO allele. CRISPR scan was used to design a gRNA sequence (5′‐GGCACGTAAAGAGGTGGAGG(GGG)‐3′). The gRNA and Cas9 mRNA synthesis and the method of embryo microinjection were carried out as described previously.^21^ To study the phenotypes of stable germline KOs of zebrafish *smc5*, we generated a frameshift mutation (c.813_814delGG, p.L271fs*8) in exon 7 of *smc5* (Figures 3B and S4). Mutation genotyping was performed by Sanger sequencing with primers: 5′‐CAAAACACACCCAGCACAAT‐3′ and 5′‐TCAGGAGGTGAAAAACAAACCTC‐3′. The tp53^−/−^ mutant allele tp53^M214K^ line^22^ was gifted by professor Thomas Look at Harvard Medical School (Boston, USA).

WT and mutant fish were obtained from WT mating and homozygous mating, respectively, except for the observation of the dwarf phenotype shown in Figure 3D. Embryos at 5‐day post‐fertilization (dpf) were captured and scored for phenotypes using a Nikon SMZ800N stereomicroscope, and body length was determined by Nikon Application Suite. Each embryo was assigned to a category depending on the SD of length for a population of WT fish from the same cohort. The numbers of surviving embryos were counted post‐fertilization for the survival curve. For rescue experiments with human SMC5 WT or R372del mutant, one‐cell‐stage smc5^−/−^ embryos were injected with 40 pg of both DNA construct tol2‐ubiquitin‐hSMC5‐2A‐EGFP and tol2 transposase mRNA. Embryos were screened at 3 dpf for the presence of GFP and the body length was scored at 5 dpf. To characterize the consequences of tp53, smc5^−/−^ embryos were injected with zebrafish tp53 MO or treated with 10‐μM Chk2 inhibitor (C3742) at 3.5–4.5 dpf, 1 or 2‐μM tp53 inhibitor, pifithrin‐α (PFT‐α) for 4 days before measuring body length at 5 dpf.

For craniofacial phenotyping, zebrafish embryos at 5 dpf were harvested, fixed overnight and stained with Alcian Blue as previously described.^23^ Phenotypes were analysed by head length and width, ceratohyal and palatoquadrate length, the distance between Meckel's cartilage and ceratohyal, and the ceratohyal angle. All experiments were carried out three times.

For whole‐mount in situ hybridization (WISH), partial cDNAs encoding zebrafish insulin, pdx1, pax6b, neurod1, pcsk1, pcsk2 and cpe were obtained by reverse transcription‐PCR with zebrafish RNA. Primers are listed in Table S7. After cloning PCR products into pGEM‐T‐easy vectors (Promega), plasmids were linearized for RNA probes using T7 transcription kits (Roche). WISH was performed and photos were taken by a Nikon SMZ25 microscope as described previously.^24^

### 2.9 Generation of Smc5^K371del^ mouse model

K371del KI mice were generated through gene editing using CRISPR/Cas9 technologies (Figure 5A). Fertilized mouse embryos were microinjected with gRNA, single‐stranded oligodeoxynucleotide containing the c.1111–1113delAAG mutation site and Cas9 mRNA and subsequently transferred into pseudopregnant C57BL6/J6 female mice. F0 founders were identified by Sanger sequencing and bred with WT mice to obtain heterozygous founder generation (SMC5^K371del/+^ or SMC5^K371/+^). Then, these heterozygous mice were mated to produce SMC5^K371/K371^ homozygous mice. Group‐housed mice were kept at 22 ± 2°C under a 12‐h light/dark cycle. Genotyping was identified with primers Smc5^371del^‐F 5′‐GATTAGTCAGCTCTTCCAGTGAGCGC‐3′ and Smc5^371del^‐R: 5′‐CGGAAACTTACTCCGTCTCTGCTTCTC‐3′. SgRNA and donor template sequences are listed in Table S8.

### 2.10 Glucose and insulin tolerance tests

Tests were performed on mice fasted for 16 and 4 h for glucose or insulin tolerance tests, respectively. After intraperitoneal injection of either 2‐g/kg glucose or .75‐U/kg insulin, the tail vein blood was collected to measure blood glucose with a glucometer (One Touch Verio Vue, Johnson & Johnson, USA) at indicated time points.

For the GTT assay in zebrafish, adult fish were starved overnight, anaesthetized with tricaine methanesulfonate (.04 mg/ml) and injected intraperitoneally with .5‐mg/body weight (g) glucose. The blood glucose was determined by the blood sample from the heart at the indicated time with a glucometer as used in mouse assays. For glucose level of embryos, 10 embryos (*n* = 8) were ground up at 5 dpf and glucose levels were measured with a glucose assay kit (BioVision), as previously described.^25^

### 2.11 Microquantitative computed tomography (microCT)

The microquantitative computed tomography (microCT) of in vivo mice was performed with a Bruker μCT (SkyScan 1176 Micro‐CT) in compliance with the standards protocols. The X‐ray source was fixed at 420 μA and 50 kVp with 1 mm for CT image acquisition. The reconstruction of the volumetric data sets was performed by the NR econ software with a modified Feldkamp algorithm. The fat tissue regions were calculated using CTAn software 1.13. Fat volume to body weight is used to compute volumetric fat percentage.

### 2.12 Immunofluorescence and immunohistochemistry (IHC)

For EdU (5‐ethynyl‐2′‐deoxyuridine) incorporation assay, seeded fibroblasts were grown with or without 250‐μM HU for 18 h and then grown in 10‐μM EdU for 30 min. EdU was detected by ‘click chemistry’ following the manufacturer's protocol (C10339, Invitrogen). Cells were examined at ×20 magnification using an Axio Scan.Z1 automatic Digital Slide Scanner (Zeiss, Germany). For each of the three experiments, a minimum of 100 cells were scored under each condition.

For γH2AX staining in MEFs, cells were treated with 1‐mM HU or 1‐mM methyl methanesulfonate (MMS, 129925, sigma) for 1 h and recovered for 24 h. Cells were stained with γ‐H2AX (Millipore, 05‐636; 1:1000) and secondary antibodies conjugated to Alexa Fluor 488 (Life Technologies) and DAPI. Images were visualized using a confocal microscope (A1, Nikon). At least 139 cells per group were counted.

For histology, fixed mouse embryos at E9.5, E15.5 or E18.5 were embedded in paraffin. After cutting the paraffin blocks into 5‐μm sections, haematoxylin and eosin (H&E) staining was carried out. The sections of tissue used for immunofluorescence or immunohistochemistry (IHC) staining were first antigen‐retrieved, blocked and then incubated overnight at 4°C with the primary antibodies as follows: Ki67 (D3B5) (Cell Signaling, 9129, 1:500), PH3 (Cell Signaling, 3548, 1:400), Ucp1 (ab10983; Abcam) and Myosin (M4276, Sigma, 1:400), γH2AX (05‐636, Millipore, 1:400), cleaved caspase‐3 (Cell Signaling, 9661,1:500). Incubation of the sections was conducted for 1 h at room temperature with secondary antibodies Alexa Fluor 488 or 594 (Life Technologies). IHC staining was performed with 3,3′‐diaminobenzidine tetrahydrochloride (Millipore). TUNEL staining of zebrafish and mouse embryos was performed using a Roche Cell Death Detection Kit (Beyotime). The zebrafish embryos were imaged with a confocal microscope (A1, Nikon) and digital images of mouse sections were obtained with a Pannoramic DESK P‐MIDI scanner (3D HISTECH). To rescue apoptosis, smc5^−/−^ embryos at 24‐h post‐fertilization (hpf) were treated with 10‐μM C3742 or 1–2‐μM PFT‐α for 12 h before TUNEL staining.

### 2.13 RNA isolation and quantitative real‐time PCR (qPCR)

The total mRNA was extracted from pools of 20 larvae or cells using TRIzol (Invitrogen) and reverse transcribed using a PrimeScript Reverse Transcriptase Kit (Takara). cDNA was analysed using SYBR Premix Ex Taq (TliRNaseH Plus) (Takara) on an ABI QuantStudio 6 Flex (Applied Biosystems). The ΔΔ*Ct* method was used for gene quantification. The primers used for quantitative real‐time PCR (qPCR) are shown in Table S7.

### 2.14 RNA‐Seq analysis

Total RNA was extracted from pools of 10 larvae with different genotyping of smc5 (WT vs. HOM) using an RNeasy minikit (Qiagen). Five biological repeats were performed for each genotype. RNA‐seq was performed on BGISEQ‐500, BGI Company (Shenzhen, China). CASAVA V1.6 package was used to process raw data. FASTQC V0.11.5 was used for sample quality control. After stringent filtering, the clean reads were aligned to the Danio rerio reference genome GRCz11 by HISAT2 software.^26^ Differential genes expression analyses (FDR < .05 and |log2FC| ≥.58) were computed by DESeq2.^27^ Gene ontology biological process (GO‐BP) and KEGG enrichment analysis were performed with Bioconductor package clusterProfiler. The analysed data are listed in Table S9.

### 2.15 Statistical analysis

GraphPad Prism software (version 9.0.0) was used to assess statistical significance through unpaired *t*‐tests with two tails and unless otherwise stated in the figure legends. *p* Values less than .05 were considered statistically significant.

## 3 RESULTS

### 3.1 A syndrome characterized by primordial dwarfism and insulin resistance

**FIGURE 1:**
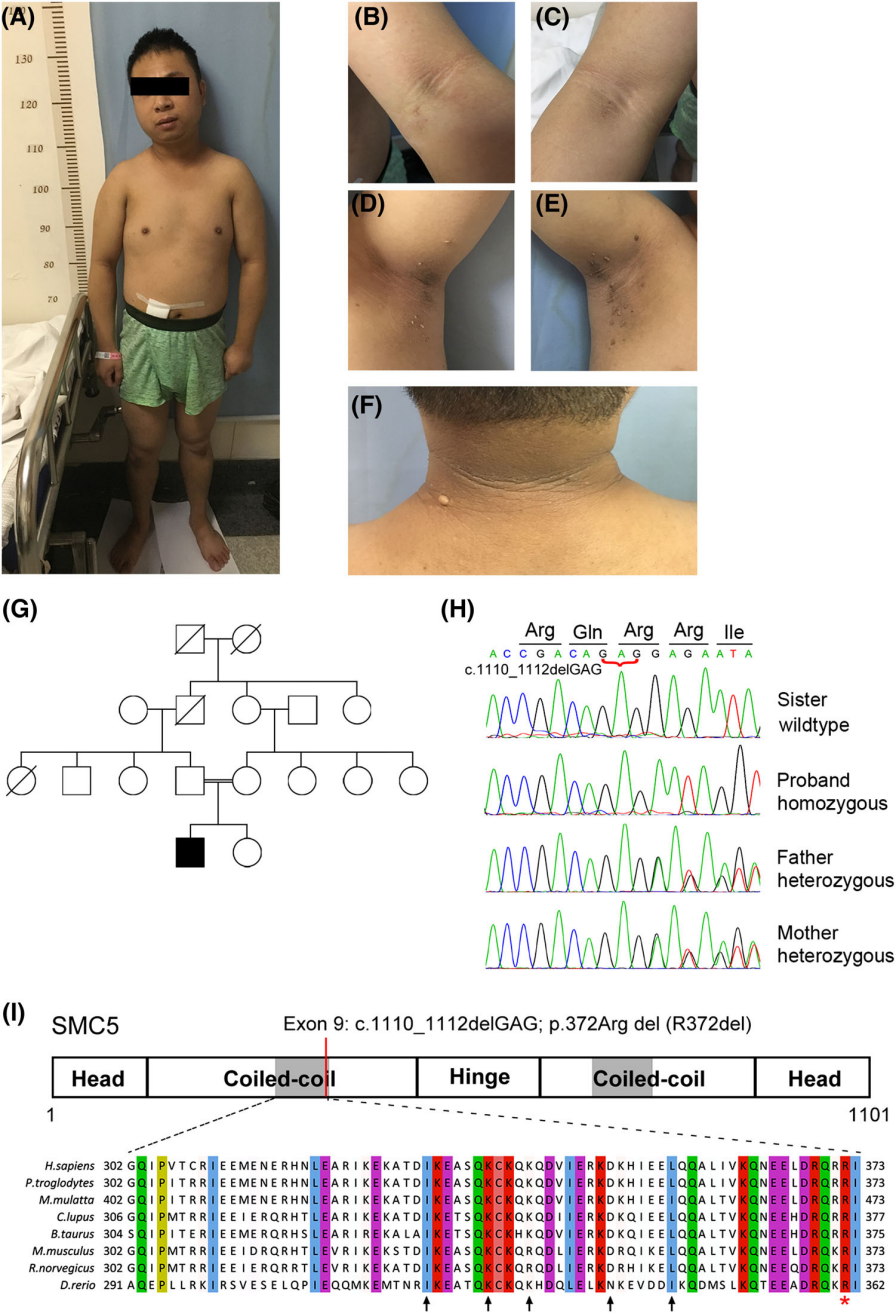
Identification of homozygous *SMC5* in‐frame deletion in the patient with primordial dwarfism and severe insulin resistance. (A–F) Clinical features in the patient included proportionate short stature (A) and severe acanthosis nigricans in the antecubital fossae (B and C), axillae (D and E) and back of the neck (F), along with skin tags. (G) Pedigree diagram of the consanguineous family. (H) Representative Sanger sequencing of the nonsense allele in the sister, the heterozygous allele in parents and the homozygous allele in patients. (I) Position of the p.Arg372del variant located in the binding domain of SMC5 (grey, p. 302–373) with the NSMCE2 protein. A multispecies alignment is shown to highlight the strong conservation of the deleted amino acid, p.Arg372 (red asterisk). Black arrows indicate SMC5 residues important for binding with NSMCE2, as described.^29^

The proband is a 29‐year‐old man of Chinese ancestry born preterm at 24 weeks of gestation in a footling breech position. Growth retardation but mild mental decline was noticed at 7–8 years of age by his parents, who were first cousins in healthy status. The appearance of acanthosis nigricans was initially noted in the neck region at approximately 10 years of age without further medical intervention. Linear growth was reported to be lifelong retardation but no evidence of osteodysplasia. He was reported to enter puberty spontaneously. Except for acanthosis nigricans and short stature, his medical history was uneventful until the first visit at age 29. On evaluation at 29‐year old, his height was 1.36 m (←3 SD), weight was 37.8 kg (←3 SD) and head circumference was 50.9 cm (←3 SD). His unaffected younger sister had normal stature (160 cm). Phenotypic findings for the proband included proportionate short stature with mild craniofacial dysmorphism, including mandibular hypoplasia and nasal prominence (Figure 1A). The absence of a distinct sloping forehead but severe acanthosis nigricans with skin tags on the antecubital fossae (Figure 1B,C), axillae (Figure 1D,E) and nape (Figure 1F) were observed. His body mass index was 21.1 kg/m^2^, and he exhibited lipoatrophy of the limbs. Abdominal magnetic resonance imaging (MRI) did not show obvious visceral or subcutaneous fat mass abnormalities (Figure S1A,B). Slightly elevated triglyceride levels (2.55 mmol/L; reference range, 0–2.25 mmol/L) correspond to mild fatty liver revealed by abdominal ultrasound. Bone turnover markers were normal, consistent with normal range of bone densitometry (Lumbar spine T score at L1–L4 was −.9; reference range, >−1).

Oral glucose tolerance tests and insulin (peptide C) releasing test were performed twice in 2016 and 2018, respectively, which all indicated elevated glucose and insulin levels at fasting and postprandial state (Table 1). The HOMA‐IR was calculated as 41.90, and HOMA‐IS as .02 based on the glucose and insulin levels (Table S1). Therefore, the proband was diagnosed with diabetes mellitus and severe insulin resistance.^28^ Metformin combined with pioglitazone (1000 mg/30 mg/day) and insulin twice daily (.5 units/kg/day) was initiated, with unexpectedly good effect. After discharge, he discontinued drug and insulin therapy. Two years later, his haemoglobin A1c levels were consistently elevated (9.4%, Table S1). Fundoscopy examinations were normal. Despite these severe biochemical features of insulin resistance, his total body fat content was just in the lower normal range detected by bioelectrical impedance analysis (4.6 kg; reference range, 4.10–9.31). Sex steroids level indicated the normal function of hypothalamic‐pituitary‐gonad axis (Table S1). The IGF‐1 (Table S1) and growth hormone stimulation test (Table S2) were normal with pituitary microadenoma on MRI (Figure S1C,D).

**TABLE 1:** Serial oral glucose tolerance test in the patient

	29 years			31 years		
Time (min)	Glucose (mmol/L)	Insulin (μU/ml)	Peptide C (ng/ml)	Glucose (mmol/L)	Insulin (μU/ml)	Peptide C (ng/ml)
0	10.1	93.35	8.50	8.5	64.95	5.79
30	17.9	119.50	9.12	17.7	56.64	6.58
60				20.6	159.30	10.37
120	24.5	208.60	13.24	18.3	218.40	12.64
180				16.2	218.70	12.29
Fasting reference range	3.9–6.1	2.6–24.9	1.1–4.4	3.9–6.1	2.6–24.9	1.1–4.4

### 3.2 Identification of an in‐frame *SMC5* variant

No significant copy number variation, neither gain nor loss, was detected in patient DNA using the Affymetrix CytoScan HD arrays. By whole‐exome sequencing, the homozygous rare nonsynonymous or splice variants in 11 genes (*SMC5, LYST, NID1, PCDH9, POLDIP2, MYH1, APBA3, PNKP, CBLB, SIDT1* and *SEZ6*; Table S3) were identified from the proband according to the filtering criteria (see Section 2), which were inherited from his heterozygous parents, respectively. Among them, a homozygous germline variant, c.1110_1112del (NM_015110; p.Arg372del) in *SMC5*, was identified in the proband, heterozygous in his parents and not found in his sibling (Figure 1G,H). This variant was not present in the dbSNP135, 1000 Genomes Project or ExAC databases. As a core component of the SMC5/6 complex, SMC5 plays an essential role in maintaining genome stability, such as neutralizing intermediates during replication and recombination, DNA damage repair via homologous recombination and chromosome segregation during mitosis and meiosis.^1^ To date, the germline mutation of the SMC5 has not been identified to be related to any phenotype in human. However, the mutations in the subunit of SMC5/6 complex, *NSMCE2*, have been reported to result in an MPD phenotype,^4^ which suggested that SMC5 might be a pathogenic gene for the patient. Moreover, the amino acid of Arg372 is highly conserved across species, located at the edge of a highly conserved N‐terminal coil‐coiled domain (Figure 1I), which is crucial for the interaction with the E3 SUMO‐ligase NSMCE2.^29^

### 3.3 Cellular consequences in patient fibroblast

**FIGURE 2:**
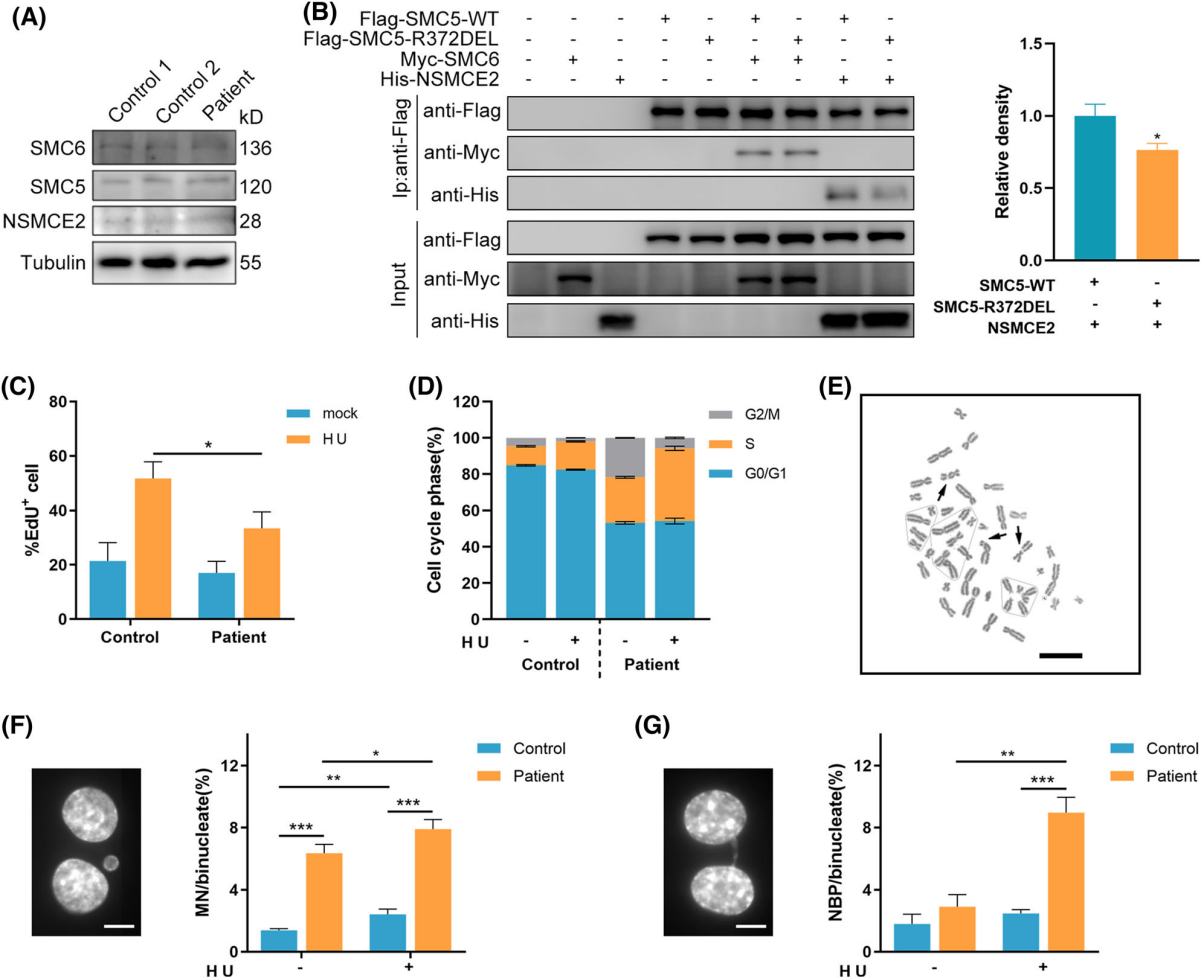
Replication stress exacerbates genome instability in patient's cells. (A) A representative western blot cropped to show unaffected NSMCE2–SMC5–SMC6 subcomplex expression in patient fibroblasts. (B) Interaction between NSMCE2 (N‐terminal HIS) or SMC6 (N‐terminal MYC) and SMC5 (N‐terminal FLAG‐tagged wild‐type (WT) or R372DEL), as determined by immunoprecipitation after transfection in HEK293T cells. Proteins were immunopurified using anti‐FLAG beads. Data are presented as mean ± s.e.m. and *n* = 5 biological replicates for quantification on the right panel. (C) HU recovery assay. Fibroblasts were incubated with mock or 250‐μM HU for 18 h, labelled with EdU for 30 min, pre‐extracted, fixed and costained for EdU and DAPI. The EdU‐positive cells were quantified. (D) Quantification of cell cycle phases in mock‐ or HU‐treated fibroblasts. *n* = 2 biological replicates were performed per condition per genotype. (E) Representative image of chromatid gaps or breaks from chromosomal aberration analysis in primary lymphocytes derived from the patient. Black arrows denote acentric fragments. Scale bar, 30 μm. (F and G) Exposure of cells to HU exacerbates micronucleus (F) and nucleoplasmic bridge (G) formation. Fibroblasts were exposed to 1‐mM HU for 4 h, followed by 24‐h recovery in the presence of 3‐μg/ml cytochalasin B, and assessed using fluorescence microscopy. In parts (C, D, F and G), data are presented as the mean ± s.d. *p < .05, **p < .01, ***p < .001 by Student's *t*‐test

In primary fibroblasts from the patient, the p.Arg372del alteration showed no effect on the endogenous protein stability of SMC5 or the complex subunits NSMCE2 and SMC6 (Figure 2A). When ectopically coexpressed Flag‐tagged WT or R372del SMC5 variant with NSMCE2 and SMC6 in 293T cells, respectively, the WT and variant SMC5 proteins showed similar expression levels and stability. However, the interaction between mutant SMC5 and NSMCE2 was ∼70% of that between WT and NSMCE2, whereas the interaction with SMC6 showed no change compared with that of the WT by coimmunoprecipitation (Figures 2B and S2). We next investigated the potential role of SMC5 deficiency in the recovery of DNA replication by incorporating of EdU. Following the release of the HU block, we observed impaired recovery of DNA replication in patient‐derived fibroblasts compared with WT (Figure 2C), accompanied by a concomitant 2–2.5‐fold increase of both basal and HU‐induced S phase arrest (Figure 2D). Chromosomal aberration analysis in lymphocytes from the patient revealed elevated cytogenetic aberrations (7%; reference range, 0–2. Figure 2E and Table S4). Furthermore, there was an approximately 3.5‐fold increase in MN and NPB formation in binucleated cells from the patient compared to controls after treatment with HU (Figure 2F,G), consistent with the levels observed in cells with *NSMCE2* or *NSMCE3* mutations.^4^, ^5^ Notably, abnormal nuclear morphology of the patient's fibroblasts was observed under electron microscopy, with enlarged nuclei, damaged nuclear membranes, decreased heterochromatin at the nuclear periphery and disorganized nuclear pore complexes (Figure S3).

### 3.4 Deficiency of smc5 resulted in shorter body length in zebrafish larvae

**FIGURE 3:**
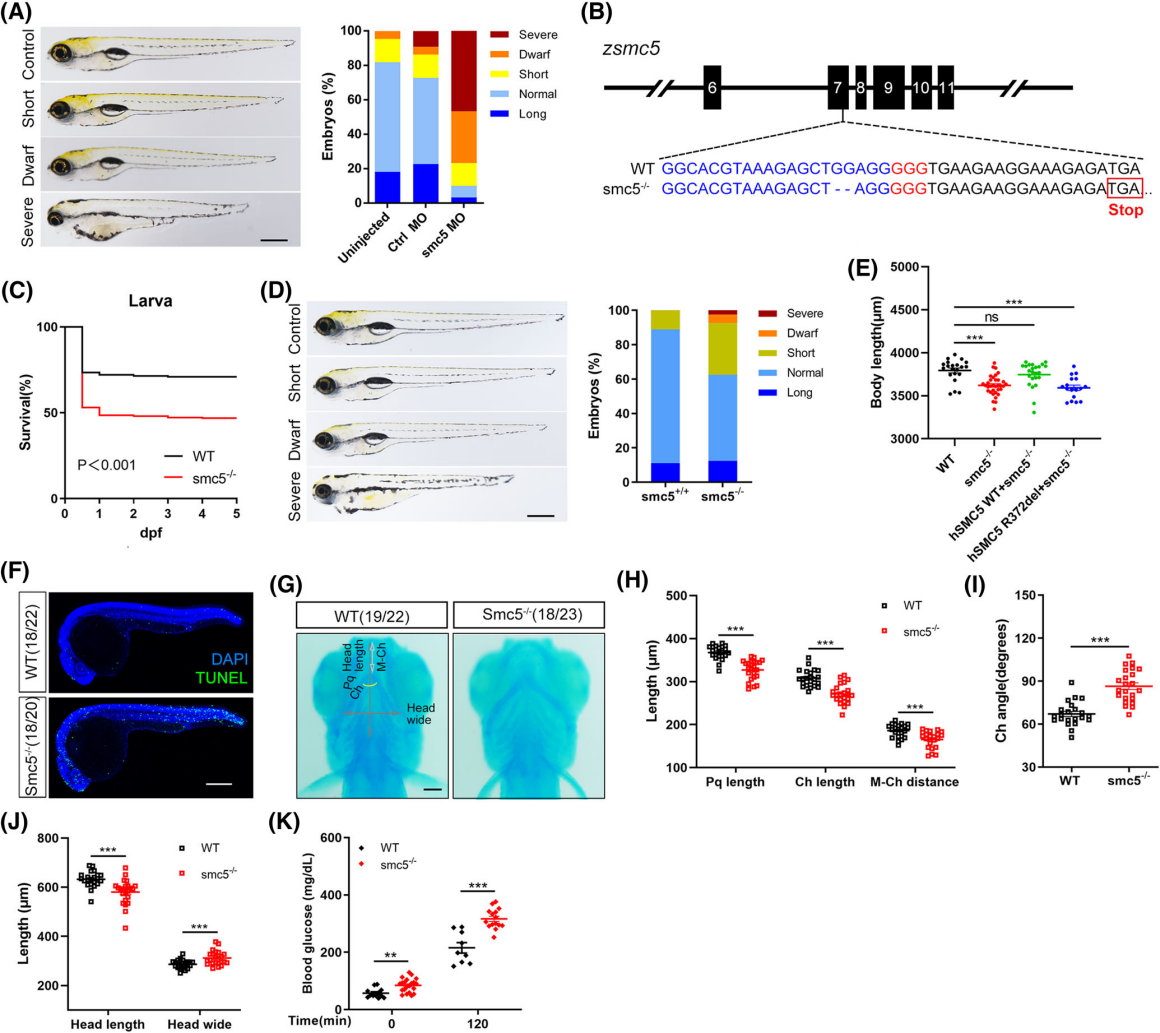
Smc5 deficiency in zebrafish led to short length, increased mortality and impaired glucose homeostasis. (A) Phenotypes and quantification of length in smc5 morpholino (MO)‐injected zebrafish. Representative images of defined length categories: long, >1 SD; normal, −1 to 1 SD; short, −1 to −2 SD; dwarf, −2 to −3 SD; severe, <−3 SD (left); and quantification of different categories (right). Uninjected, *n* = 22; control MO, *n* = 22; smc5 MO, *n* = 30. Scale bar = 500 μm. (B) CRISPR target site in exon 7 of zebrafish smc5 (zsmc5) gene, resulting in 2‐bp deletion allele and premature stop codons (red box). The CRISPR‐guided RNA sequence and the protospacer adjacent motif (PAM) are highlighted in blue and red, respectively. (C) Survival curve over time from wild‐type (WT) and smc5^−/−^ larvae. *n* = 950 and 962 for WT, smc5^−/−^ larvae, respectively; *p* < .001. Log‐rank Mantel–Cox test. (D) Phenotypes and quantification of length in smc5 knockout (KO) zebrafish recorded as in (A). Smc5^+/+^, *n* = 36; smc5^−/−^, *n* = 40. Scale bar = 500 μm. (E) Coinjection of human SMC5 tol2 plasmid (WT or R372del) with transposase mRNA and body length was scored at 5 dpf. WT, *n* = 21; smc5^−/−^, *n* = 34; hSMC5 WT+smc5^−/−^, *n* = 24; hSMC5 R372del+smc5^−/−^, *n* = 17. (F) TUNEL staining showed numerous apoptotic cells in the brain and posterior segment of spinal cord in smc5^−/−^ embryos at 24 hpf. Scale bar = 250 μm. (G) Representative ventral images of Alcian blue staining of cartilage structures show severe defects in smc5^−/−^ larvae at 5 dpf. Pq, palatoquadrate; Ch, ceratohyal arch; M–Ch, distance from Meckel's cartilage (M) to Ch; the yellow curve indicates Ch angle. WT, *n* = 22; smc5^−/−^, *n* = 23. Scale bars = 100 μm. (H–J) Quantitative analysis of a series of changes of phenotypic indexes, including Pq, Ch and M–Ch length (H), Ch angle (I) and head length and wide (J) in G. (K) The glucose tolerance test in 3‐month‐old WT and smc5^−/−^ zebrafish. Blood glucose was measured at 0 (WT, *n* = 12; smc5^−/−^, *n* = 21) and 120 min (WT, *n* = 9; smc5^−/−^, *n* = 15) after glucose injection. Values represent the mean ± s.e.m. **p < .01, ***p < .001 by Student's *t*‐test

The SMC5 protein is well conserved, with orthologous genes readily detectable in multiple species. The human SMC5 is 90% identical to its mouse orthologue and 57% identical to its zebrafish orthologue. Zebrafish has one reciprocal orthologue of smc5 located on chromosome 10, encoded by 1073 amino acids. To evaluate the pathogenic effects of SMC5 loss of function in vivo, we first knock down the *smc5* gene by MO in zebrafish embryos and result in significantly shorted body length. It produced a defect phenotype, of which 77% of embryos exhibited severe growth restriction or dwarf phenotypes, compared with 14% in control MO‐injected embryos (Figure 3A). Then we established smc5 KO zebrafish by CRISPR‐Cas9, with a 2‐bp deletion in smc5 exon 7 (Figures 3B and S4). Homozygous smc5 KO zebrafish are viable and fertile; however, only ∼ 50% of homozygous embryos survived (Figure 3C). Furthermore, the size of homozygous embryos that survived was markedly reduced at 5 dpf, with a significantly increased percentage of dwarf and severely growth restricted larvae. About 2.5% of homozygous embryos displayed an extreme phenotype, such as cardiac oedema and increased mortality (Figure 3D). Coinjection of a Tol2 plasmid containing human WT SMC5 cDNA with transposase mRNA efficiently attenuated the short phenotype observed in smc5^−/−^ embryos at 5 dpf. In contrast, coinjection of R372del variant identified in the patient failed to rescue the smc5 KO phenotype (Figure 3E). These findings suggest that smc5 is crucial for embryonic survival and/or growth in zebrafish and the SMC5 R372del variant identified in the patient lead to loss of function. Using TUNEL staining, we observed marked apoptosis in the brain and spinal cord in smc5 KO embryos compared with age‐matched WT embryos (Figure 3F), coinciding with the slow longitudinal growth described earlier. Consistent with that the patient showed mild craniofacial dysmorphism, a group of craniofacial cartilage defects were found in smc5 KO larvae (Figure 3G), including a shorter length of ceratohyal arch (Ch), palatoquadrate (Pq) and distance from Meckel's cartilage (M) to Ch (Figure 3H) and enlarged Ch angle (Figure 3I), which may contribute to a shorter but broader head (Figure 3J). Interestingly, *smc5* KO did not alter glucose levels and pancreatic endocrine differentiation and/or maturation markers as well as insulin expression in 5‐dpf embryos (Figure S5). However, elevated glucose levels were observed in smc5 KO adults at 3‐month post‐fertilization (Figure 3K).

**FIGURE 4:**
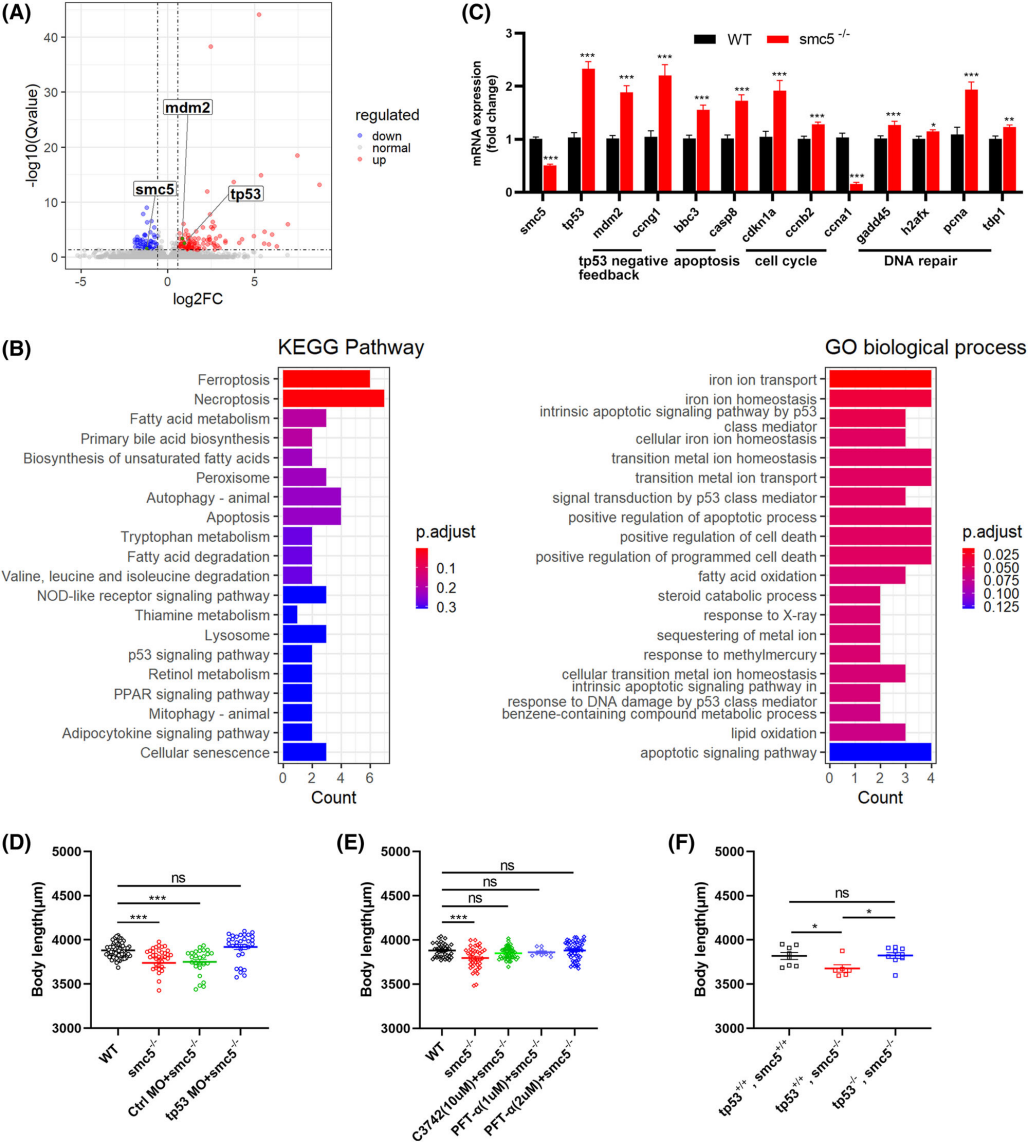
Smc5 deficiency activates tp53‐related apoptosis pathway in zebrafish. (A) Volcano plot showing common differentially expressed genes (DEGs) of HOM and wild‐type (WT) 3‐dpf embryos. The horizontal line indicates a log10 (adjusted *p* value) of 1.3 (Qvalue of .05); and the vertical lines a log2 fold change of −.58 and .58. (B) Top 20 enriched KEGG pathway and gene ontology (GO) biological process (ranked by p.adjust) from DEGs as in (A) using the ClusterProfiler R package. (C) The expression of genes involved in the DNA damage response and signal transduction by *tp53* detected in 3‐dpf larvae by quantitative real‐time PCR (qPCR). Actin was chosen as the internal reference gene. (D) Knockdown of *tp53* partially rescued the short phenotype in smc5^−/−^ larvae. WT, *n* = 77; smc5^−/−^, *n* = 36; smc5^−/−^ + Ctrl morpholino (MO), *n* = 26; smc5^−/−^ + tp53 MO, *n* = 31; (E) body length of smc5^−/−^ embryos at 5‐dpf treated with 10‐μM C3742 from 3.5 to 4.5 dpf or indicated concentration of PFT‐α from 24 hpf to 5 dpf. WT, *n* = 38; smc5^−/−^, *n* = 38; C3742 (10 μM) + smc5^−/−^, *n* = 46; PFT‐α (1 μM) + smc5^−/−^, *n* = 9; PFT‐α (2 μM) + smc5^−/−^, *n* = 46; (F) deletion of *tp53* in smc5 knockout (KO) zebrafish rescued the reduced body length. tp53^+/+^, smc5^+/+^, *n* = 9; tp53^+/+^, smc5^−/−^, *n* = 6; tp53^−/−^, smc5^−/−^, *n* = 9. In parts (C–F), values represent the mean ± s.e.m. *p < .05, **p < .01, ***p < .001

To investigate the global effects of *smc5* KO on gene expression, we performed RNA‐seq analysis on RNA obtained from WT and smc5 KO larvae harvested at 3 dpf. A total of 173 differentially expressed genes (105 upregulated and 68 downregulated genes) were identified (Figure 4A and Table S9). The top KEGG pathway and GO‐BP term enrichment analysis indicated a cellular response centred on the p53 signalling pathway (Figure 4B). The KEGG enrichment pathways contained ferroptosis, apoptosis and cellular senescence, and the GO‐BP term contained apoptotic process response to DNA damage and cell death. Meanwhile, these targets participate in lipid metabolism and other important biological processes, which were likely caused by a self‐protection during stress in response to smc5 deficiencies. p53 Activation is the most common apoptosis‐inducing pathway,^30^, ^31^ and we tested whether this pathway is involved in the apoptosis of *smc5* KO zebrafish by qPCR. Accordingly, the expression of *tp53* and its downstream genes involving tp53 regulation (*mdm2, ccng1*), apoptosis *(bbc3, casp8)*, cell cycle (*cdkn1a, ccnb2*) and DNA repair (*gadd45, h2afx, pcna, tdp1*) showed significant upregulation in smc5 mutants (Figure 4C). Furthermore, knockdown *tp53* by MO injecting in smc5^−/−^ zebrafish could effectively rescue its short body length observed (Figure 4D). We further tried chemical inhibitors C3742 and PFT‐α, which are kinase inhibitors of chk2 and tp53, respectively. As expected, both inhibitors can partially rescue shorted body length and increased apoptosis in smc5^−/−^ zebrafish (Figures 4E and S6). In fact, the deletion of *tp53* in smc5^−/−^ zebrafish could effectively increase the body length (Figure 4F, Table S10), consistent with a recent report of SMC5 cKO mice with microcephalus.^32^, ^33^ Together, these results suggested that activation of the p53 pathway is the direct cause of dwarf phenotype in smc5^−/−^ larvae.

### 3.5 Smc5*^K371/K371^* mice showed reduced survival rates and adiposity

**FIGURE 5:**
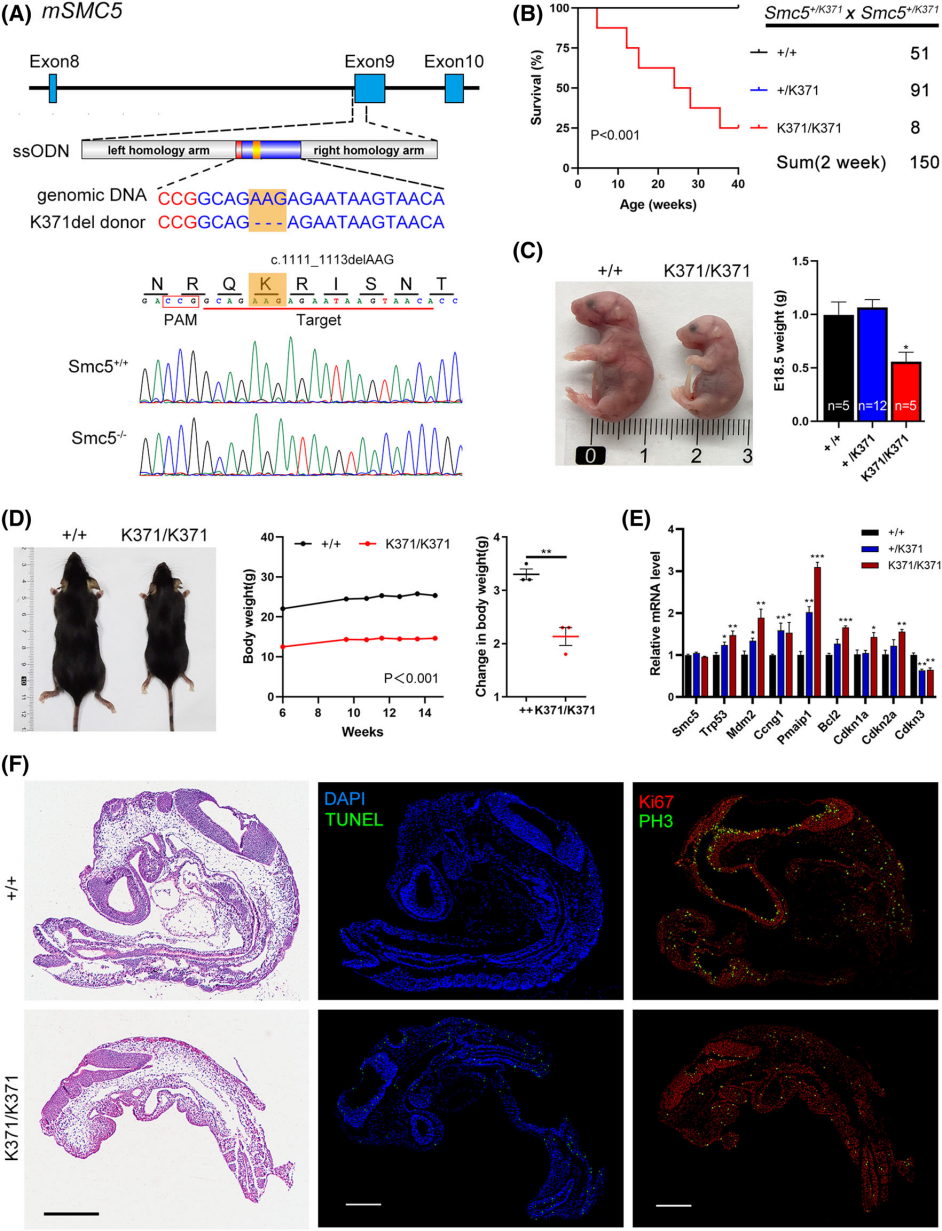
*Smc5^K371del^* knock‐in mice are small and have a higher embryonic lethality rate. (A) Scheme of the knock‐in strategy for CRISPR/Cas9‐mediated deletion of K371 in mice. The single‐stranded oligodeoxynucleotide (ssODN) was designed with arm sequences and the target site, among which the site for K371del is shown in detail. The protospacer adjacent motif (PAM) site is shown in red, the sgRNA site is shown in blue, and K371 is boxed in orange squares, indicating the point mutation (c.1111_1113delAAG, p.K371del) (upper panel). Sanger sequencing of genomic DNA isolated from mice with the indicated genotypes was shown in the lower panel. (B) Survival curve and genotypes of offspring from Smc5^+/K371^ intercrosses. *p* < .001. Log‐rank Mantel–Cox test. (C) Images and weights of embryos at E18.5. (D) Representative photograph of male mice at 15 weeks of age (left), body weight (middle) and body weight gain after feeding a chow diet for 8 weeks (right) (*n* = 3). (E) The expression of Trp53‐related genes involved in apoptosis and cell cycle detected in mouse embryonic fibroblasts (MEFs) by quantitative real‐time PCR (qPCR). Rplp0 (36B4) was chosen as the internal reference gene. (F) Sections of embryos at E9.5 stained with haematoxylin and eosin (H&E) (scale bar = 500 μm), costained for TUNEL and DAPI, and Ki67 and PH3, respectively (scale bar = 200 μm). In parts (C–E), values represent the mean ± s.e.m. *p < .05, **p < .01, ***p < .001

Considering early embryonic lethality observed in complete SMC6 or NSMCE2 KO mice,^34^, ^35^ we generated CRISPR KI K371del mutant mice (Figure 5A). As the amino acid corresponding to human smc5 c.1110_1112del can be considered arginine deletion at position 371 or 372 (defined as 372 in this study), an Smc5^K371del^ mouse model was generated in which lysine at position 371 was deleted, but arginine at position 372 (same as in humans) was retained. Homozygous KI mice (Smc5*^K371del/K371del^*, hereafter referred to as Smc5*^K371/K371^*) were born but at a much lower rate than expected frequency of Mendelian heredity, suggesting a remarkably decreased survival rate both in utero and ex utero (Figure 5B). At E18.5, the *Smc5^K371/K371^* embryos were smaller and weighed significantly less than the WT embryos (Figure 5C), indicating that defects in Smc5 impair vitality and fitness during embryonic development. After birth, Smc5*^K371/K371^* mice appeared to grow in size but remained far smaller than their littermates, exhibiting an overall slower growth rate and much lower body weight (Figure 5D). As expected, MEFs from Smc5*^K371/K371^* mice showed an upregulation of p53 and its downstream genes (Figure 5E), similar to those in *smc5* KO larvae. We then analysed WT and Smc5^K371/K371^ MEFs for DNA damage by examining levels of γH2AX. As shown in Figure S7, the amount of γH2AX was slightly higher in the Smc5^K371/K371^ cells than that in WT cells under basal condition. However, following HU and MMS treatment, Smc5^K371/K371^ cells displayed much higher levels of γH2AX compared to WT cells. E15.5 Smc5^K371/K371^ embryos also showed an increase in the proportion of γH2AX‐positive cells (Figure S8). DNA damage was detectable in all tissues but was particularly evident in embryonic brain of Smc5^K371/K371^ embryos. Moreover, IHC staining for cleaved caspase‐3 revealed that Smc5^K371/K371^ embryos contained an increased number of apoptotic cells (Figure S8). Accordingly, immunostaining and TUNEL in E9.5 *Smc5^K371/K371^* embryos showed reduced Ki67^+^ and pH3^+^ (phosphorylated histone H3) cells and increased apoptosis signal, especially in the region of cerebromedullary tube (Figure 5F). These data indicate that the K371del variant causes DNA damage throughout the embryo, which ultimately results in increased apoptosis and failure of the embryos to develop normally.

**FIGURE 6:**
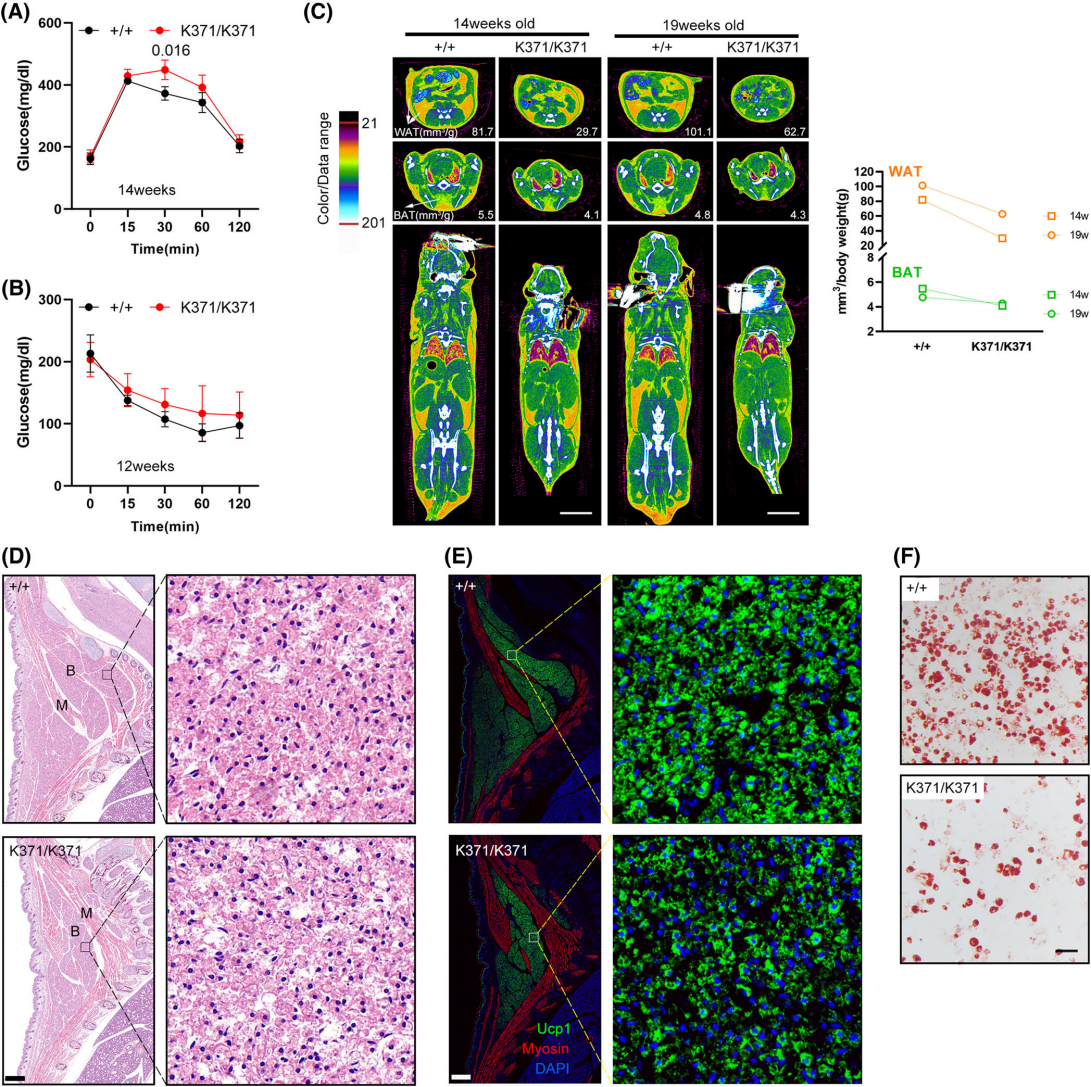
Reduced adiposity in Smc5^K371/K371^ mice. (A) Glucose levels (assessed by an intraperitoneal glucose tolerance test) at 14 weeks of age were significantly higher in K371/K371 mice than in wild‐type (WT) control mice at the 30‐min time point. (B) In an insulin tolerance test, glucose was reduced to a lesser extent trend in K371/K371 mice than in their WT control counterparts at 12 weeks of age. Pairwise significance compared to controls is shown. In parts (A and B), *n* = 3 for each group the analysis used two‐way ANOVA with repeated measures and Bonferroni correction (GraphPad Prism 6). All error bars are plotted as a mean value ± s.e.m. (C) Representative two‐dimensional cross‐sectional μCT images in transverse planes of the abdomen (top), scapular (middle) and coronal sections of the whole body (bottom) showing white and brown adipose tissue. Scale bars = 10 mm. The fat volume was quantified and shown in the right panel. (D and E) Histological analysis of E18.5 embryos. Sagittal sections of the cervical/thoracic area were stained with haematoxylin and eosin (H&E) (D) or with antibodies against the brown adipose (B) marker UCP1 (green) and the muscle (M) marker Myosin (red) (E). Scale bar = 400 μm. Boxed regions in each image were magnified and showed details of embryonic brown adipose precursors. Lipid droplets can be seen forming immaturely in the mutants. (F) Oil red O staining of WT and mutant mouse embryonic fibroblasts (MEFs) after 7 days of white adipogenesis. Scale bar = 100 μm

Notably, homozygous mice displayed a pattern of impaired glucose tolerance (Figure 6A) with mild alteration of insulin sensitivity on standard normal chow diet (Figure 6B). In addition, we observed reduced percentage of total fat using microCT analysis, including both white and brown adipose tissue (Figure 6C), which was also observed in Nsmce2‐deficient adult mice.^35^ It is now universally acknowledged that white and brown adipocytes originate from distinct precursor cells. We then explored whether Smc5 mutation affects fat development as early as embryonic stage. Brown adipose precursor cell pools are easily visible by H&E staining of sectioned embryos. Smc5 mutation decreases the size of brown fat precursor cell pools in Smc5*^K371/K371^* E18.5 embryos (Figure 6D,E), consistent with the impact of K371 variant for limiting DNA damage and apoptosis (Figure S8). At high magnification, E18.5 mutant precursors immaturely begin accumulating lipid (Figure 6D) with reduced UCP1 expression (Figure 6E), suggesting that aberrant lipid accumulation is secondary to a primary defect in cell growth. Unlike brown adipose formation, subcutaneous begins to develop perinatally, whereas visceral fat is formed postpartum. We, therefore, used MEFs as progenitors to induce white adipogenesis. The Smc5*^K371/K371^* MEFs differentiated to adipocyte‐like cells but with significantly reduced efficiency compared to MEFs from WT littermates (Figure 6F), underlying the support for a role of SMC5 in adipogenesis.

### 3.6 SMC5 is required for adipocyte differentiation in vitro

**FIGURE 7:**
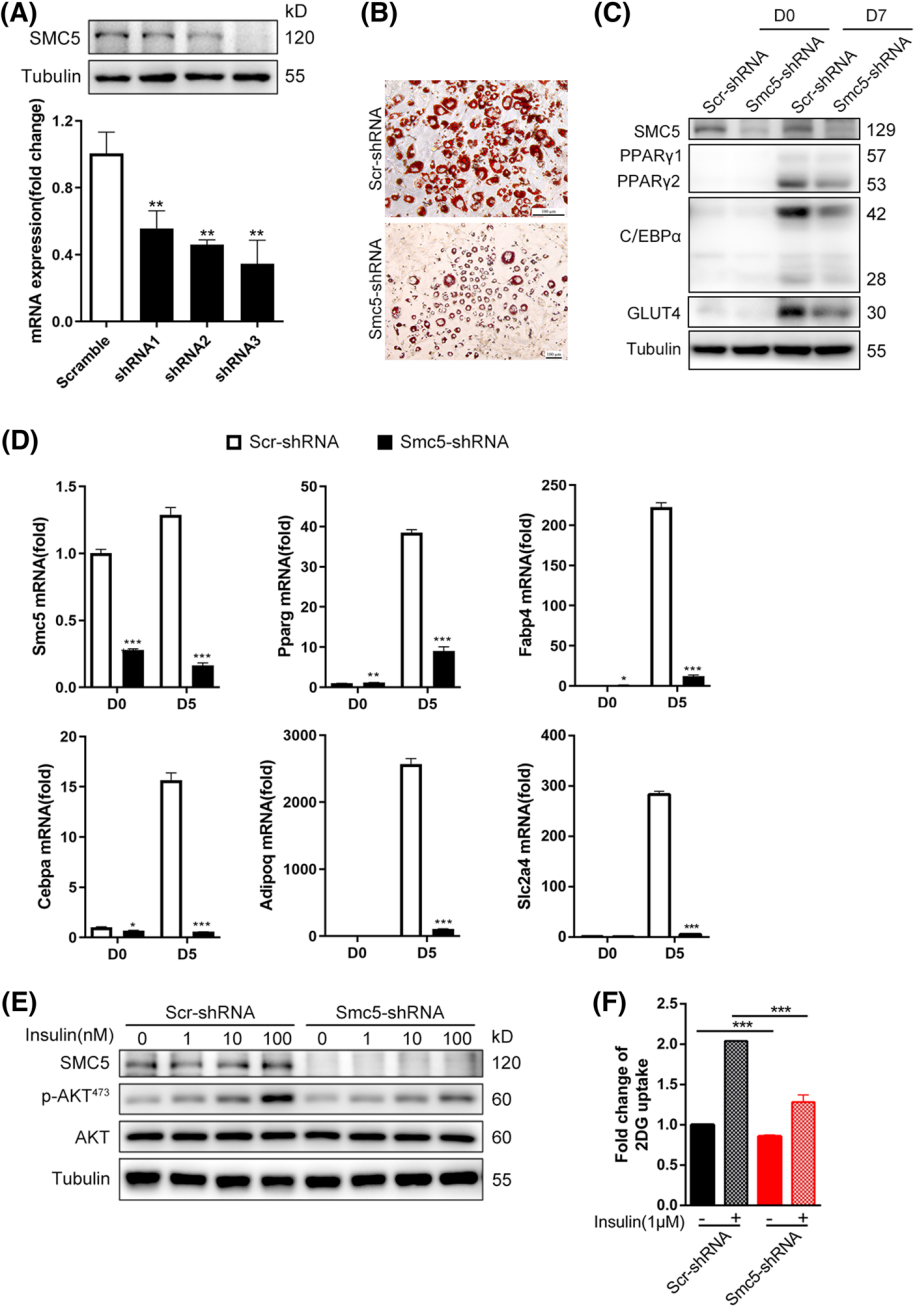
Smc5 is required for 3T3‐L1 adipogenesis. (A) The 3T3‐L1 white preadipocytes were infected with lentivirus shRNAs targeting Smc5 (Smc5‐shRNA) or scramble (Scr‐shRNA) virus, followed by adipogenesis assay. Quantitative real‐time PCR (qPCR) and Western blot confirmation of Smc5 knockdown efficiency before adipogenesis. Values represent the mean ± s.d. (B) Oil red O staining at D7 of adipogenesis. Scale bars = 30 μm. (C) Western blots of PPARγ, C/EBPα and GLUT4 in preadipocytes (D0) and adipocytes (D7). (D) qPCR of Pparg, Cebpa, Fabp4, Adipoq, Slc2a4 expression at D0 and D5 of adipogenesis. Values represent the mean ± s.e.m. (E) Western blots of phosphorylated Akt at Serine 473 and total Akt in protein extracts of adipocytes (D7). (F) Insulin‐stimulated glucose uptake assessed by the 2‐deoxyglucose assay in adipocytes (D7). Values represent the mean ± s.d. In parts (A and D), Rplp0 (36B4) was chosen as the internal reference gene. For parts (A, D and F), the *p* values were calculated using a two‐sided Student's *t*‐test. *p < .05, **p < .01 and ***p < .001

To explore the mechanism of insulin resistance and glucose metabolism disorder, in vitro shRNA assay was performed in hepatocyte cell lines HepG2 and 3T3‐L1 preadipocytes. Unexpectedly, qPCR showed reduced expression of G6PC, PCK1, FBP1 with the down‐regulation of SMC5, suggesting that hepatic gluconeogenesis is not the cause of hyperglycaemia (Figure S9). Then the cell‐autonomous role of Smc5 in adipogenesis was explored. It was revealed that knockdown of Smc5 significantly affected the differentiation of adipocytes, accompanied by decreased expression of the key adipocyte regulators PPARγ, CEBPα and GLUT4 (Figure 7A–D). As a result, reduced AKT phosphorylation at Serine 473 (Figure 7E) and that at glucose uptake (Figure 7F) were observed in Smc5‐knockdown 3T3‐L1 adipocytes, indicating reduced insulin sensitivity.

**FIGURE 8:**
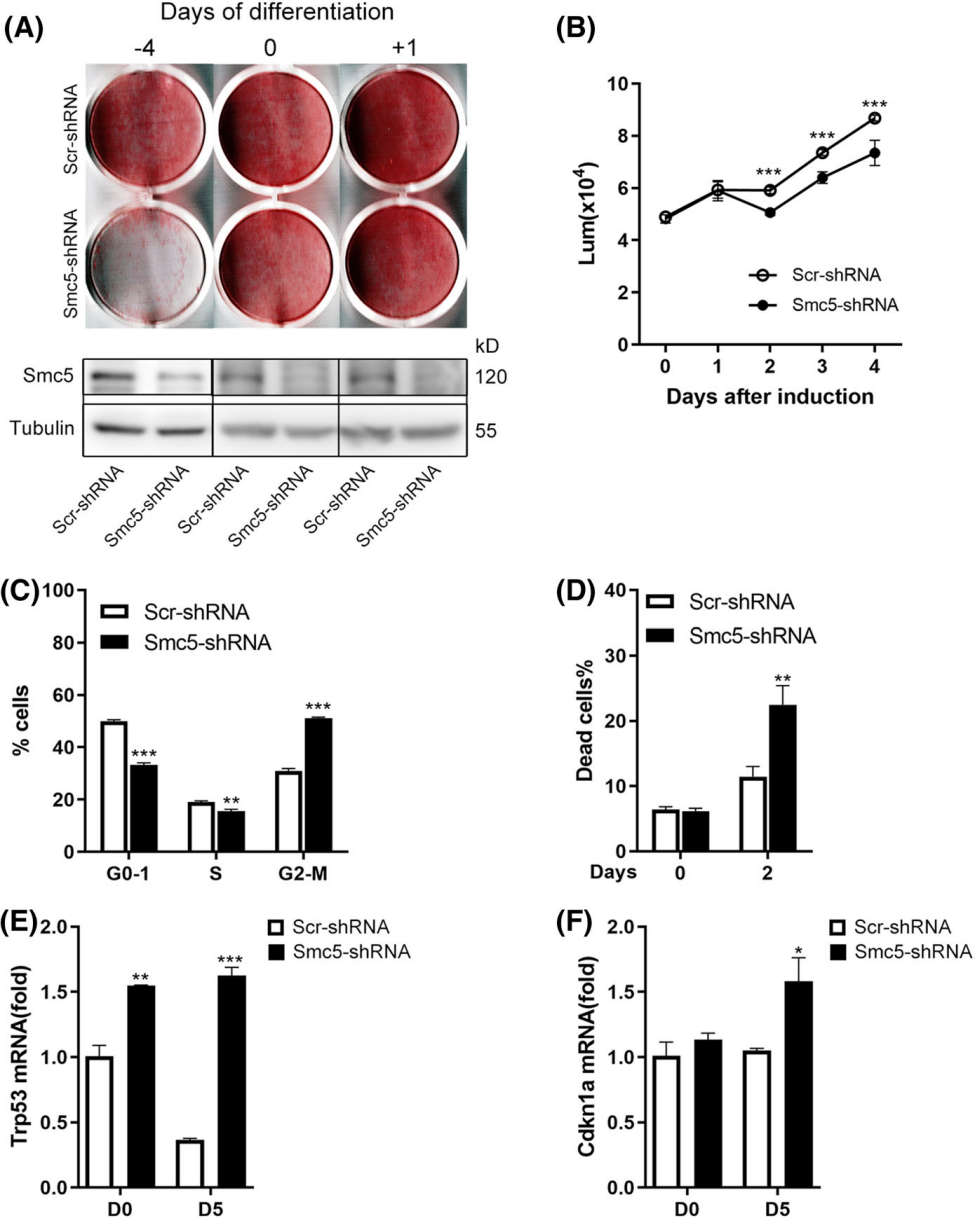
Smc5 is essential for mitotic clonal expansion during adipogenesis. (A) Representative stained plates (top) and Western blots (bottom) of 3T3‐L1 cells infected with Smc5 or scrambled shRNA at different time points, followed by induction of adipogenesis. (B) Relative proliferation of 3T3‐L1 white preadipocyte cells before and after differentiation was assessed by CellTiter‐Glo assay, *n* = 5. (C) Cell cycle profile by fluorescence‐activated cell sorting (FACS) at D2 of adipogenesis. (D) Quantification of dead cells (7‐AAD) by FACS at D0 and D2 of adipogenesis. (E and F) Quantitative real‐time PCR (qPCR) of Trp53 (p53) and Cdkn1a (p21) expression at D0 and D5 of adipogenesis. Rplp0 (36B4) was chosen as the internal reference gene. The data are presented as the mean ± s.d. in (B–D) and mean ± s.e.m. in (E and F). For parts (B–F), the *p* values were calculated using a two‐sided Student's *t*‐test. *p < .05, **p < .01 and ***p < .001

Interfering with the expression of Smc5 led to severe defect in adipogenesis, which was observed when the lentivirus infection occurred 4 days before differentiation (D − 4). Knockdown of Smc5 at D0 partially blocked adipogenesis, whereas knockdown at D1 did not affect it (Figure 8A). These data suggest that Smc5 is required for adipogenesis at the early stage of clonal expansion. Then, the growth curve during adipogenesis was evaluated considering the role of SMC5/6 complex in mitosis, and we evaluated the growth curve during adipogenesis. Smc5 deficiency inhibited mitotic clonal expansion (MCE) (Figure 8B), which could be partially explained by G2/M phase arrest (Figure 8C) and increased cell death at day 2 of differentiation (Figure 8D). The activation of p53 has been shown to be essential for adipocyte differentiation, proliferation and survival.^36^ *p53* and *Cdkn1a* expression increased in Smc5‐knockdown cells before and after differentiation compared with scramble‐knockdown cells (Figure 8E,F), suggesting that Smc5 downregulation could interfere with MCE mediated by activating the p53 pathway, resulting in impaired adipogenesis.

## 4 DISCUSSION

Members of the SMC family are essential for cell proliferation and differentiation, as well as maintaining chromosome structure and integrity.^37^ Human SMC5/6 consists of two core SMC proteins, SMC5 and SMC6, and six non‐SMC elements. Our study demonstrates the association between homozygous in‐frame deletion of Arg372 in SMC5 and a syndrome characterized by primordial dwarfism, extreme insulin resistance and diabetes.

Mutations within genes encoding SMC complex components are associated with developmental defects in humans, but insulin resistance has rarely been investigated in these disorders. Growth retardation, craniofacial anomalies, microbrachycephaly and reduced body fat have been described in cohesinopathies such as Cornelia de Lange syndrome CdLS, which is inherited in an autosomal dominant (NIPBL, SMC2 or RAD21) or X‐linked (SMC1A or HDAC8) pattern.^38^ The gene expression profiling analysis in the Nipbl^+/−^ mouse model suggested alterations in adipogenesis.^39^, ^40^ Very rare cases with mutations in condensin components were reported to exhibit primary microcephaly due to failure of chromosome decatenation during mitosis.^41^ The SMC5/6 complex has been revealed to stabilize and restart stalled replication forks, maintaining replication through highly repetitive regions of the genome, which is essential for mitotic growth in human cells.^42^ In the two genomic instability syndromes caused by mutations in non‐SMC elements of SMC5/6, the NSMCE2 mutations cause primordial dwarfism, extreme insulin resistance and gonadal failure,^4^ whereas the NSMCE3 mutations lead to lung disease immunodeficiency and chromosome breakage syndrome in early childhood.^5^ Consistent with the patient cells harbouring the NSMCE2 mutation, the assessment of our patient cells with SMC5 Agr372del demonstrated chromosome instability and increased sensitivity to DNA replication stress. Elevated MN and NPB formation in binucleated cells was observed in the patient's fibroblasts treated with HU, as well as elevated cytogenetic aberrations in the lymphocytes. However, the potential link between defects in the SMC5/6 complex and insulin resistance remains unclear.

In accordance with the findings in primordial dwarfism of Majewski type 2 caused by pericentrin (PCNT) mutation and in Werner syndrome caused by WRN mutation,^43^ severe insulin resistance is not congenital in these patients but appears during late childhood or adolescence. Diabetes could develop at the adult stage with progressive loss of limb adipose tissue. Huang‐Doran et al.^44^ also found that all patients identified with PCNT mutation younger than 4‐year old did not present with insulin resistance. Moreover, our patient showed a mild phenotype with a relatively later onset due to the in‐frame deletion of *SMC5*, which only partially affected the interaction of SMC5 and NSMCE2 without interfering the stability of the SMC5/6 complex. The occurrence of late‐onset diabetes in our patient and diabetes that aggravated with age due to NSMCE2 mutations^4^ suggests that metabolic abnormalities caused by defective Smc5/6 complex function may be age‐related.

Compared with WT littermates, *Smc5^K371/K371^* mice showed obviously smaller and constant lower body weight, which was related with significantly reduced fat mass as revealed by microCT. Indeed, Smc5^K371/K371^ mice showed similar glucose change and fat loss as SMC6‐S994A^34^ and NSMCE2 cKO^35^ mice. Maintenance of systemic insulin sensitivity requires normal size and function of key insulin‐responsive tissues, such as adipose. The severe insulin resistance in our patient is reminiscent of lipodystrophy, which is primarily characterized by a complete or partial loss of adipose tissue and impaired lipid storage capacity. However, the relationship between SMC5/6 complex and adipogenesis has not been investigated clearly. It is well known that MCE is a synchronous process required for adipogenesis, when the growth‐arrested 3T3‐L1 preadipocytes in G1 phase synchronously re‐enter the cell cycle and undergo two rounds of mitosis then exit the cell cycle and commence terminal differentiation into adipocytes.^45^ Blocking MCE also impairs the DNA binding activity of C/EBPβ, which initiates a transcriptional cascade of C/EBPα and PPARγ, interfering with the terminal differentiation of adipocyte.^46^ Defects of adipogenesis were observed in the *Smc5^K371/K371^* MEFs, also proved in 3T3‐L1 cells with Smc5 knockdown. Furthermore, the impaired adipogenesis was revealed to be caused by G2/M phase arrest during the process of MCE. Accordingly, in mouse embryonic stem cells with a conditional KO of Smc5, Pryzhkova et al.^47^ found that the downregulation of Smc5 results in cells accumulating in the G2 phase. Moreover, mitotic defects are apparent only when the function of SMC5/6 is compromised during the S/G2 phases, suggesting its crucial role in the sister chromatid disjunction.^7^

In the adipocyte, insulin signalling is critical for glucose uptake induced by insulin. In Smc5 downregulated adipocytes, reduced expression of adipocyte markers (*PPARγ, C/EBPα*, etc.) was observed, accompanied with decreased GLUT4 and phosphorylated Akt, leading to impaired insulin‐stimulated glucose uptake assessed by the 2‐deoxyglucose assay. Interestingly, PCNT knockdown in preadipocytes also impaired insulin‐stimulated glucose uptake, demonstrating an associated defect in cell proliferation, adipogenesis and insulin sensitivity.^44^

P53 plays an integrating and pivotal role in coordinating cellular reactions to stress signals, mainly by activating cell‐cycle arrest, apoptosis, or senescence.^48^ However, the mechanisms by which p53 coordinates metabolism on the organ level remain poorly understood.^49^ The elevated levels of apoptosis were revealed in the embryos of KO zebrafish and KI mice, which resulted from the upregulation of p53. Knockdown or KO tp53 partially restores the body length and apoptosis in the smc5 KO zebrafish, consistent with the result in Smc5 cKO mice with microcephalus.^32^, ^33^ Dysregulation of P53 was demonstrated to be involved in abnormal adipogenesis. Consistently, adipocyte differentiation defects were revealed in the Smc5‐depleted preadipocytes. A mildly elevated level of postprandial blood glucose and reduced insulin sensitivity was found in the *Smc5* KI mice. However, due to low breeding efficiency and high embryonic mortality rates, replicating the entire metabolic phenotype in mice has proven insurmountable. Further study will be needed to assess the metabolic phenotypes in extended observation windows with extreme caloric loads, or in tissue‐specific Smc5 KO mice.

In summary, we identified a homozygous in‐frame deletion of Arg372 in the *SMC5* gene as the cause of a syndrome of MPD, diabetes and extreme insulin resistance. In vitro analysis indicated that Arg372 deletion at the edge of the coil‐coiled domain impaired the interaction between SMC5 and the E3 SUMO ligase NSMCE2. Our result was supported by the dwarfism and hyperglycaemia phenotypes in smc5^−/−^ zebrafish; more interestingly, it was further supported by the reduced survival rate, severely shortened body size and lipodystrophy in the K371del knock‐in mouse model.

## FUNDING INFORMATION

This work was supported by the grants from the National Natural Science Foundation of China (Grant nos. 81873652, 81770786), National Key R&D Program of China (Grant no. 2017YFC1001801) and Shanghai Municipal Natural Science Foundation (Grant no. 21ZR1438300).

## CONFLICT OF INTEREST

The authors declare no competing interests.

## Supporting information

Supporting Information

Supporting Information

## Data Availability

The data that support the findings of this study are available from the corresponding author upon reasonable request.

## References

[1] Diaz M , Pecinka A . Scaffolding for repair: understanding molecular functions of the SMC5/6 complex. Genes. 2018;9(1):36. 29329249 10.3390/genes9010036 PMC5793187

[2] Kanno T , Berta DG , Sjogren C . The Smc5/6 complex is an ATP‐dependent intermolecular DNA linker. Cell Rep. 2015;12(9):1471‐1482. 26299966 10.1016/j.celrep.2015.07.048

[3] Menolfi D , Delamarre A , Lengronne A , Pasero P , Branzei D . Essential roles of the Smc5/6 complex in replication through natural pausing sites and endogenous DNA Damage tolerance. Mol Cell. 2015;60(6):835‐846. 26698660 10.1016/j.molcel.2015.10.023 PMC4691243

[4] Payne F , Colnaghi R , Rocha N , et al. Hypomorphism in human NSMCE2 linked to primordial dwarfism and insulin resistance. J Clin Invest. 2014;124(9):4028‐4038. 25105364 10.1172/JCI73264 PMC4151221

[5] van der Crabben SN , Hennus MP , McGregor GA , et al. Destabilized SMC5/6 complex leads to chromosome breakage syndrome with severe lung disease. J Clin Invest. 2016;126(8):2881‐2892. 27427983 10.1172/JCI82890 PMC4966312

[6] Aragon L . The Smc5/6 complex: new and old functions of the enigmatic long‐distance relative. Annu Rev Genet. 2018;52:89‐107. 30476445 10.1146/annurev-genet-120417-031353

[7] Venegas AB , Natsume T , Kanemaki M , Hickson ID . Inducible degradation of the human SMC5/6 complex reveals an essential role only during interphase. Cell Rep. 2020;31(3):107533. 32320646 10.1016/j.celrep.2020.107533

[8] Goss CM . Bird‐headed dwarfs—studies in developmental anthropology including human proportions. By Helmut P. G. Seckel. xiii + 241 pages, 64 figures. $10.00. Charles C Thomas, Publisher, Springfield. 1960. Anat Rec. 1961;139(1):92‐92.

[9] Majewski F , Ranke M , Schinzel A . Studies of microcephalic primordial dwarfism II: The osteodysplastic type II of primordial dwarfism. Am J Med Genet. 1982;12(1):23‐35. 7201238 10.1002/ajmg.1320120104

[10] Melvin A , O'Rahilly S , Savage DB . Genetic syndromes of severe insulin resistance. Curr Opin Genet Dev. 2018;50:60‐67. 29477938 10.1016/j.gde.2018.02.002

[11] Zhang LL , Kan M , Zhang MM , et al. Multiregion sequencing reveals the intratumor heterogeneity of driver mutations in TP53‐driven non‐small cell lung cancer. Int J Cancer. 2017;140(1):103‐108. 27646734 10.1002/ijc.30437

[12] Li H , Durbin R . Fast and accurate short read alignment with Burrows‐Wheeler transform. Bioinformatics. 2009;25(14):1754‐1760. 19451168 10.1093/bioinformatics/btp324 PMC2705234

[13] DePristo MA , Banks E , Poplin R , et al. A framework for variation discovery and genotyping using next‐generation DNA sequencing data. Nat Genet. 2011;43(5):491‐498. 21478889 10.1038/ng.806 PMC3083463

[14] Li H , Handsaker B , Wysoker A , et al. The sequence alignment/map format and SAMtools. Bioinformatics. 2009;25(16):2078‐2079. 19505943 10.1093/bioinformatics/btp352 PMC2723002

[15] Wang K , Li M , Hakonarson H . ANNOVAR: functional annotation of genetic variants from high‐throughput sequencing data. Nucleic Acids Res. 2010;38(16):e164. 20601685 10.1093/nar/gkq603 PMC2938201

[16] Adzhubei IA , Schmidt S , Peshkin L , et al. A method and server for predicting damaging missense mutations. Nat Methods. 2010;7(4):248‐249. 20354512 10.1038/nmeth0410-248 PMC2855889

[17] Wang N , Zhu W , Han B , et al. Inherited missense mutation occurring in arginine76 of the SRY gene does not account for familial 46, XY sex reversal. J Clin Endocrinol Metab. 2020;105(5):dgaa109. 32140723 10.1210/clinem/dgaa109

[18] Zufferey R , Nagy D , Mandel RJ , Naldini L , Trono D . Multiply attenuated lentiviral vector achieves efficient gene delivery in vivo. Nat Biotechnol. 1997;15(9):871‐875. 9306402 10.1038/nbt0997-871

[19] Liu D , Zhang P , Zhou J , et al. TNFAIP3 interacting protein 3 overexpression suppresses nonalcoholic steatohepatitis by blocking TAK1 activation. Cell Metab. 2020;31(4):726‐740.e728. 32268115 10.1016/j.cmet.2020.03.007

[20] Agency IAE . Cytogenetic dosimetry: applications in preparedness for and response to radiation emergencies. Vienna: IAEA. 2011;232.

[21] Sun F , Fang Y , Zhang MM , et al. Genetic manipulation on zebrafish duox recapitulate the clinical manifestations of congenital hypothyroidism. Endocrinology. 2021;162(8):bqab101. 34019632 10.1210/endocr/bqab101

[22] Berghmans S , Murphey RD , Wienholds E , et al. tp53 mutant zebrafish develop malignant peripheral nerve sheath tumors. Proc Nat Acad Sci USA. 2005;102(2):407‐412. 15630097 10.1073/pnas.0406252102 PMC544293

[23] Lee YR , Khan K , Armfield‐Uhas K , et al. Mutations in FAM50A suggest that Armfield XLID syndrome is a spliceosomopathy. Nat Commun. 2020;11(1):3698. 32703943 10.1038/s41467-020-17452-6 PMC7378245

[24] Yang RM , Zhan M , Zhou QY , et al. Upregulation of GBP1 in thyroid primordium is required for developmental thyroid morphogenesis. Genet Med. 2021;23(10):1944‐1951. 34194003 10.1038/s41436-021-01237-3 PMC8486662

[25] Gut P , Baeza‐Raja B , Andersson O , et al. Whole‐organism screening for gluconeogenesis identifies activators of fasting metabolism. Nat Chem Biol. 2013;9(2):97‐104. 23201900 10.1038/nchembio.1136 PMC3552031

[26] Kim D , Paggi JM , Park C , Bennett C , Salzberg SL . Graph‐based genome alignment and genotyping with HISAT2 and HISAT‐genotype. Nat Biotechnol. 2019;37(8):907‐915. 31375807 10.1038/s41587-019-0201-4 PMC7605509

[27] Love MI , Huber W , Anders S . Moderated estimation of fold change and dispersion for RNA‐seq data with DESeq2. Genome Biol. 2014;15(12):550. 25516281 10.1186/s13059-014-0550-8 PMC4302049

[28] Gambineri A , Zanotti L , Ibarra‐Gasparini D . Androgens and severe insulin resistance states: basic and clinical aspects. Front Horm Res. 2019;53:177‐186. 31499491 10.1159/000494911

[29] Duan X , Sarangi P , Liu X , Rangi GK , Zhao X , Ye H . Structural and functional insights into the roles of the Mms21 subunit of the Smc5/6 complex. Mol Cell. 2009;35(5):657‐668. 19748359 10.1016/j.molcel.2009.06.032 PMC2993495

[30] Aylon Y , Oren M . Living with p53, dying of p53. Cell. 2007;130(4):597‐600. 17719538 10.1016/j.cell.2007.08.005

[31] Vousden KH . p53: death star. Cell. 2000;103(5):691‐694. 11114324 10.1016/s0092-8674(00)00171-9

[32] Atkins A , Xu MJ , Li M , Rogers NP , Pryzhkova MV , Jordan PW . SMC5/6 is required for replication fork stability and faithful chromosome segregation during neurogenesis. eLife. 2020;9:e61171. 33200984 10.7554/eLife.61171 PMC7723410

[33] Phan TP , Maryniak AL , Boatwright CA , et al. Centrosome defects cause microcephaly by activating the 53BP1‐USP28‐TP53 mitotic surveillance pathway. EMBO J. 2020;40:e106118. 33226141 10.15252/embj.2020106118 PMC7780150

[34] Ju L , Wing J , Taylor E , et al. SMC6 is an essential gene in mice, but a hypomorphic mutant in the ATPase domain has a mild phenotype with a range of subtle abnormalities. DNA Repair (Amst). 2013;12(5):356‐366. 23518413 10.1016/j.dnarep.2013.02.006

[35] Jacome A , Gutierrez‐Martinez P , Schiavoni F , et al. NSMCE2 suppresses cancer and aging in mice independently of its SUMO ligase activity. Nucleic Acids Res. 2015;34(21):2604‐2619. 10.15252/embj.201591829 PMC4641528 26443207

[36] Lee YK , Chung Y , Lee JH , Chun JM , Park JH . The intricate role of p53 in adipocyte differentiation and function. Cells. 2020;9(12). 10.3390/cells9122621 PMC7762213 33297294

[37] Wu N , Yu H . The Smc complexes in DNA damage response. Cell Biosci. 2012;2:5. 22369641 10.1186/2045-3701-2-5 PMC3329402

[38] Piché J , Van Vliet PP , Pucéat M , Andelfinger G . The expanding phenotypes of cohesinopathies: one ring to rule them all!. Cell Cycle. 2019;18(21):2828‐2848. 31516082 10.1080/15384101.2019.1658476 PMC6791706

[39] Sarogni P , Pallotta MM , Musio A . Cornelia de Lange syndrome: from molecular diagnosis to therapeutic approach. J Med Genet. 2020;57(5):289‐295. 31704779 10.1136/jmedgenet-2019-106277 PMC7231464

[40] Kawauchi S , Calof AL , Santos R , et al. Multiple organ system defects and transcriptional dysregulation in the Nipbl(+/‐) mouse, a model of Cornelia de Lange Syndrome. PLos Genet. 2009;5(9):e1000650. 19763162 10.1371/journal.pgen.1000650 PMC2730539

[41] Martin CA , Murray JE , Carroll P , et al. Mutations in genes encoding condensin complex proteins cause microcephaly through decatenation failure at mitosis. Genes Dev. 2016;30(19):2158‐2172. 27737959 10.1101/gad.286351.116 PMC5088565

[42] Palecek JJ . SMC5/6: multifunctional player in replication. Genes. 2018;10(1):7. 30583551 10.3390/genes10010007 PMC6356406

[43] Oshima J , Sidorova JM . Werner syndrome: clinical features, pathogenesis and potential therapeutic interventions. Ageing Res Rev. 2017;33:105‐114. 26993153 10.1016/j.arr.2016.03.002 PMC5025328

[44] Huang‐Doran I , Bicknell LS , Finucane FM , et al. Genetic defects in human pericentrin are associated with severe insulin resistance and diabetes. Diabetes. 2011;60:925‐935. 21270239 10.2337/db10-1334 PMC3046854

[45] Tang QQ , Otto TC , Lane MD . Mitotic clonal expansion: a synchronous process required for adipogenesis. Proc Nat Acad Sci USA. 2003;100(1):44‐49. 12502791 10.1073/pnas.0137044100 PMC140878

[46] Tang QQ , Lane MD . Activation and centromeric localization of CCAAT/enhancer‐binding proteins during the mitotic clonal expansion of adipocyte differentiation. Genes Dev. 1999;13(17):2231‐2241. 10485846 10.1101/gad.13.17.2231 PMC316997

[47] Pryzhkova MV , Jordan PW . Conditional mutation of Smc5 in mouse embryonic stem cells perturbs condensin localization and mitotic progression. J Cell Sci. 2016;129(8):1619‐1634. 26919979 10.1242/jcs.179036 PMC4852767

[48] Olive KP , Tuveson DA , Ruhe ZC , et al. Mutant p53 gain of function in two mouse models of Li‐Fraumeni syndrome. Cell. 2004;119(6):847‐860. 15607980 10.1016/j.cell.2004.11.004

[49] Lacroix M , Riscal R , Arena G , Linares LK , Le Cam L . Metabolic functions of the tumor suppressor p53: implications in normal physiology, metabolic disorders, and cancer. Mol Metabol. 2020;33:2‐22. 10.1016/j.molmet.2019.10.002 PMC7056927 31685430

