# Supplementary material for: In‐frame deletion of SMC5 related with the phenotype of primordial dwarfism, chromosomal instability and insulin resistance
Source: Clin Transl Med. 2023 Jan 10;13(1):e1007. doi: 10.1002/ctm2.1007 (PMC9832215; doi:10.1002/ctm2.1007)
Supplement: Supplementary file 1 — Supporting Information [file CTM2-13-e1007-s001.pdf]

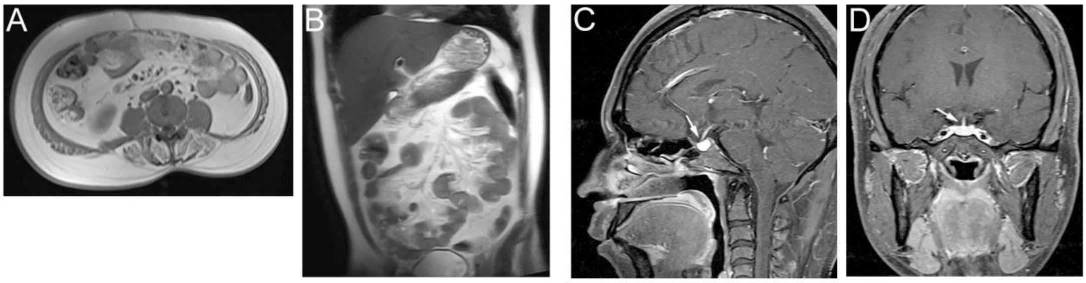

Figure S1: MRI. (A, B) Representative fat distribution in abdominal MRI showing no obvious abnormality of intraabdominal and subcutaneous fat. (A) Axial T1-weighted image at level L3. (B) Sagittal T1-weighted image. (C, D) Sagittal (C) and coronal (D) contrast-enhanced T1-weighted MRI showing pituitary microadenomas (indicated by arrows).

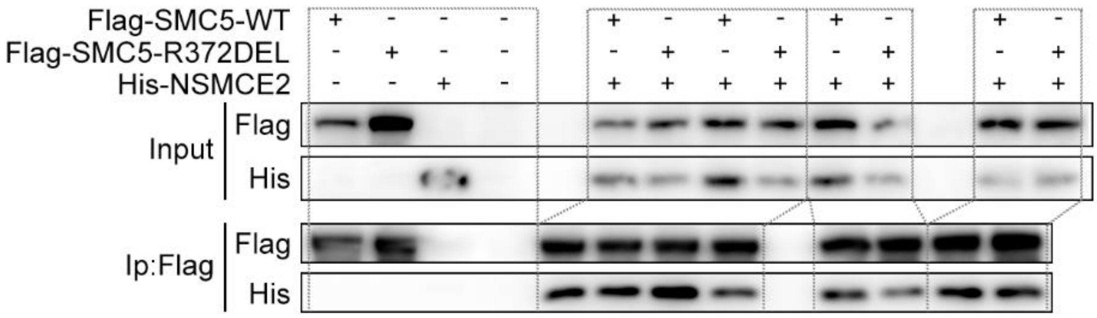

Figure S2 (Related to Figure 2B): SMC5 mutation slightly reduced its interaction with NSMCE2. Co-IP analysis of the interaction between NSMCE2 and SMC5 in 293T transfected by SMC5-WT-Flag/ SMC5-R372DEL-Flag and NSMCE2-His. Proteins were immunopurified using anti-FLAG beads.

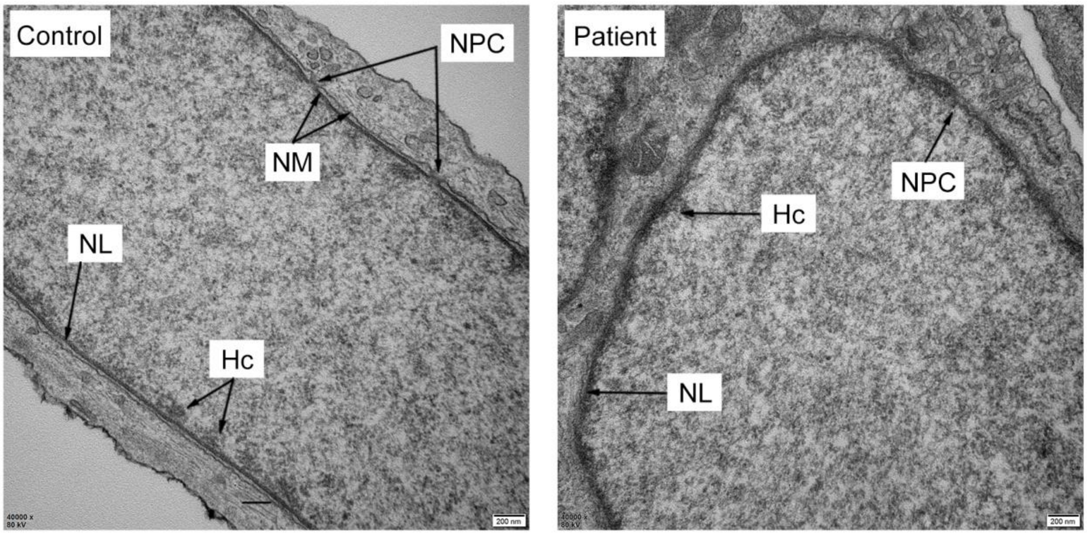

Figure S3: Electron microscopy. Electron micrographs of patient fibroblasts show nuclear membrane (NM) damage, decreased heterochromatin (Hc) at the nuclear periphery, disorganized nuclear pore complex (NPC) and dilated nuclear lamina (NL). Scale bar = 200 nm.

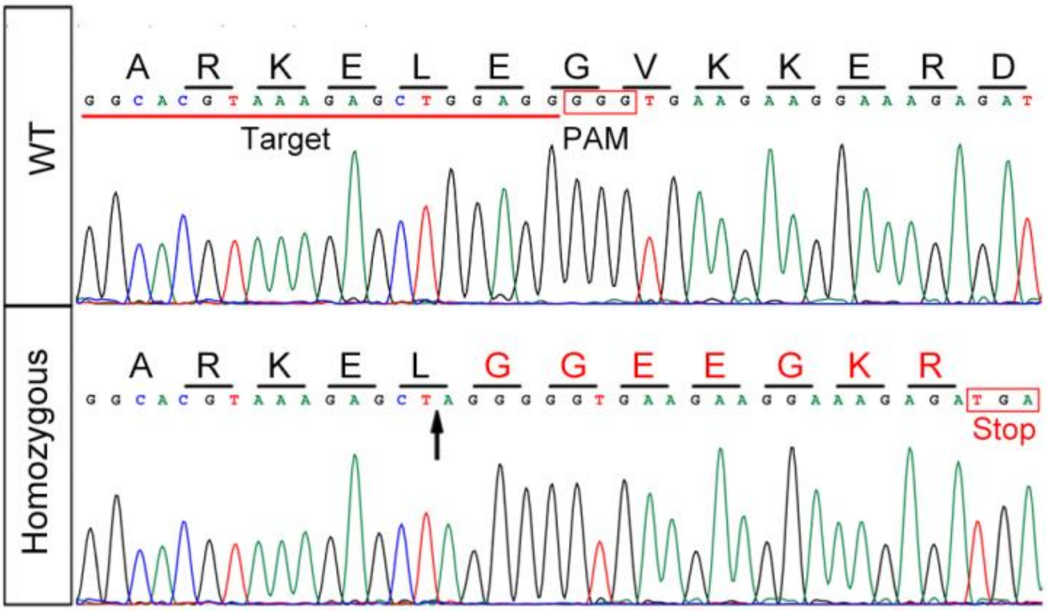

Figure S4 (Related to Figure 3B): Sanger sequencing of zebrafish mutation alleles. smc5 knockout allele was created by introducing a frameshift mutation (c.813_814delGG, p.L271fs*8). The black arrow indicates the deletion position.

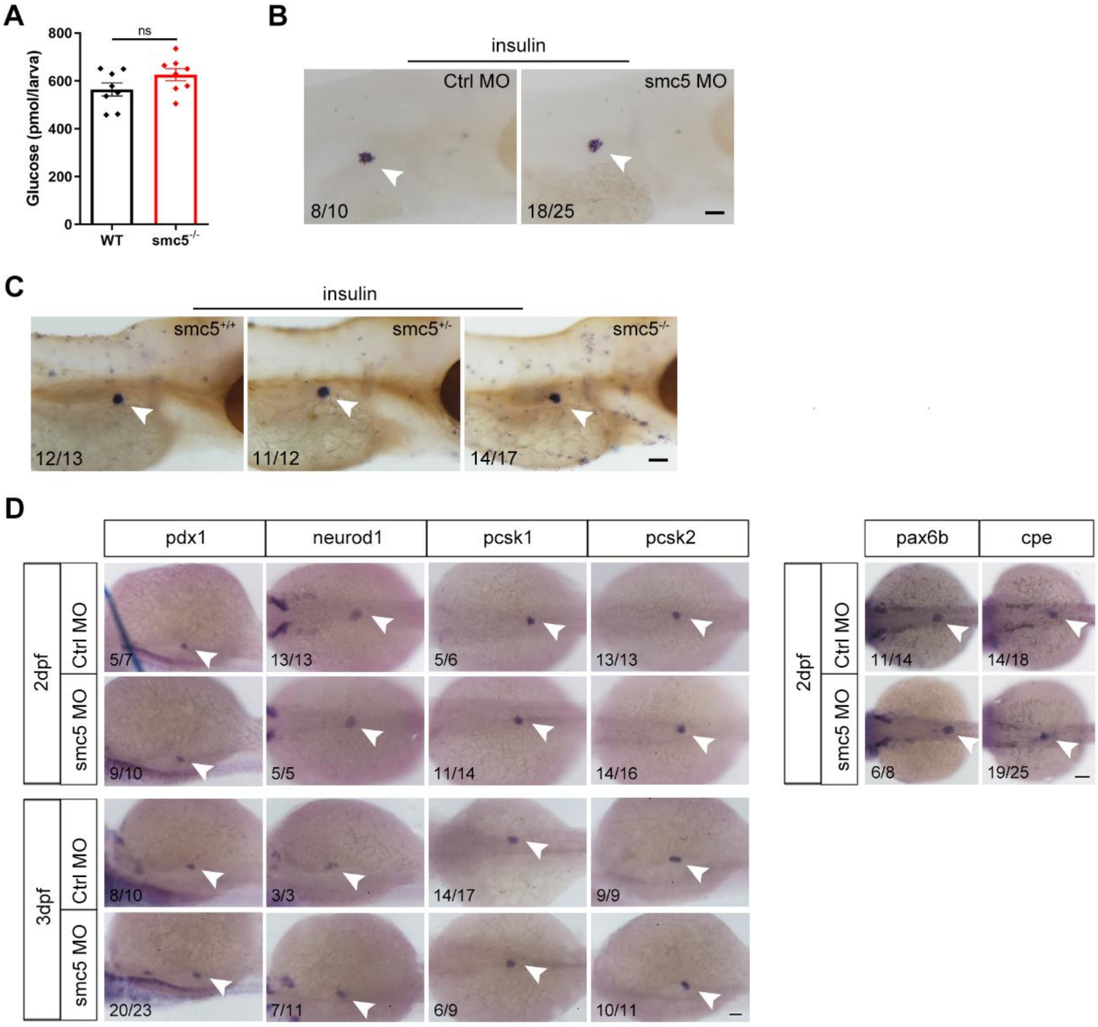

Figure S5: SMC5 deficiency has no effect on glucose level and pancreatic endocrine differentiation and/or maturation markers in 5dpf embryos. (A) Glucose levels from 5 dpf whole larval extracts (pools of 10 embryos, n=8). (B-C) WISH analysis of insulin expression in control- and smc5 MO- injected embryos (B) and embryos from smc5$^{+/-}$ intercrosses (C) at 5 dpf, arrows. Scale bar = 1mm. (D) WISH analysis of indicated endocrine genes (pdx1, neurod1, pax6b) and proinsulin processing enzymes (pcsk1, pcsk2, cpe) expression in control - and smc5 MO - injected embryos at 5 dpf, arrows. On the left panel, the scale bar = 100um. On the right panel, the scale bar = 1mm.

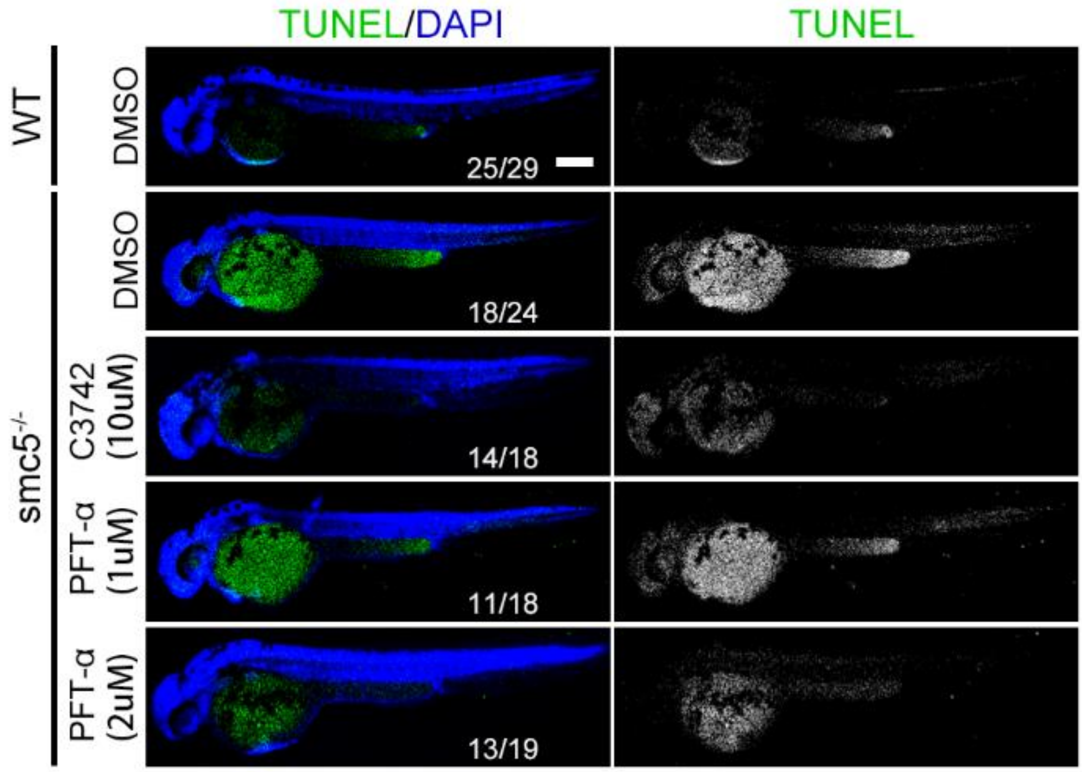

Figure S6: Inhibition of P53 signaling alleviated apoptotic signals in smc5$^{-/-}$ embryos. TUNEL staining of 36 hpf embryos treated with 10uM C3742 or indicated concentration of PFT-α from 24 hpf to 36 hpf. Scale bar = 200 µm.

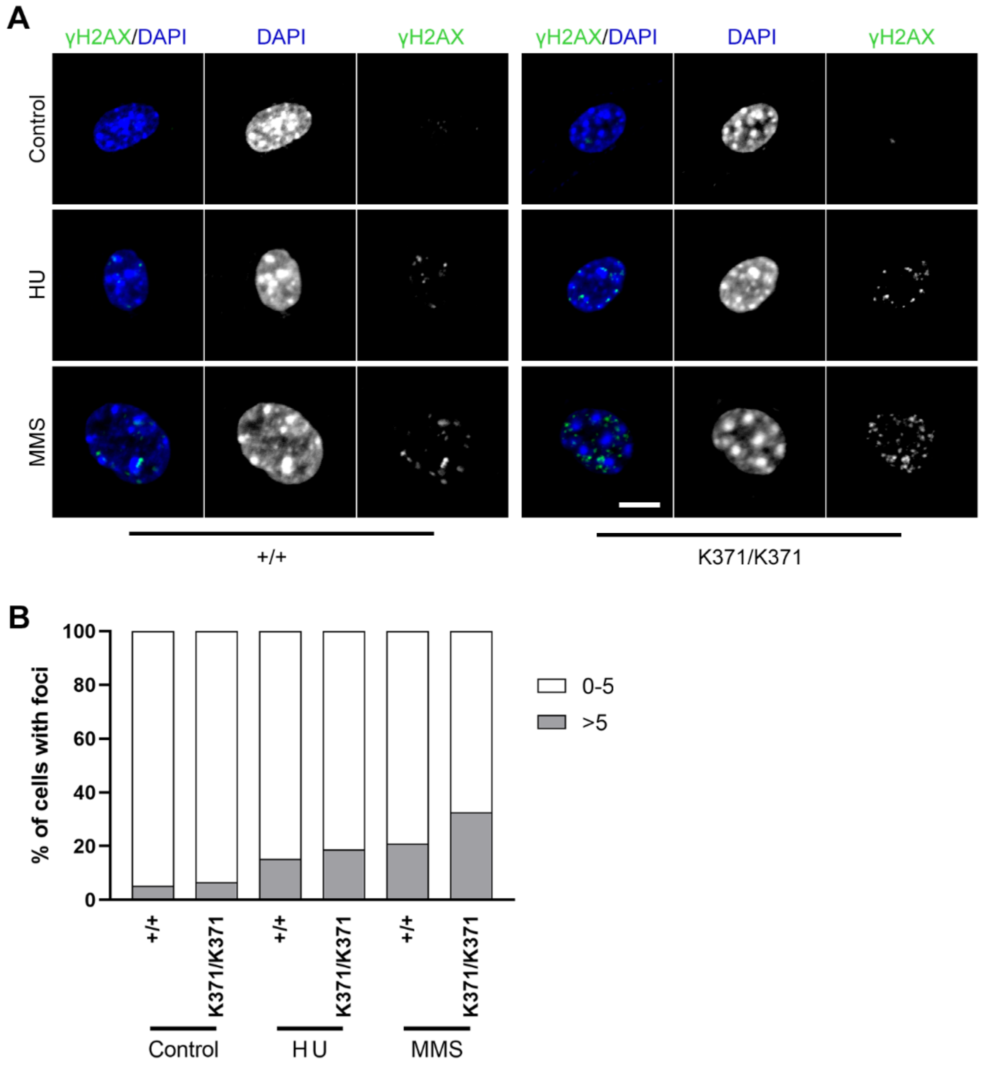

Figure S7: Increased levels of DNA damage in Smc5$^{K371/K371}$ cells. (A) Representative immunofluorescence confocal microscopy images of γH2AX immunofoci recruitment in response to 1 mM HU and 1 mM MMS (1 h and 24 h recovery)-induced DNA damage in Smc5$^{K371/K371}$ MEFs. Scale bar = 10 µm. A representative image for each condition is shown. (B) Quantification of γH2AX foci for each group in A. At least 139 cells per group were counted.

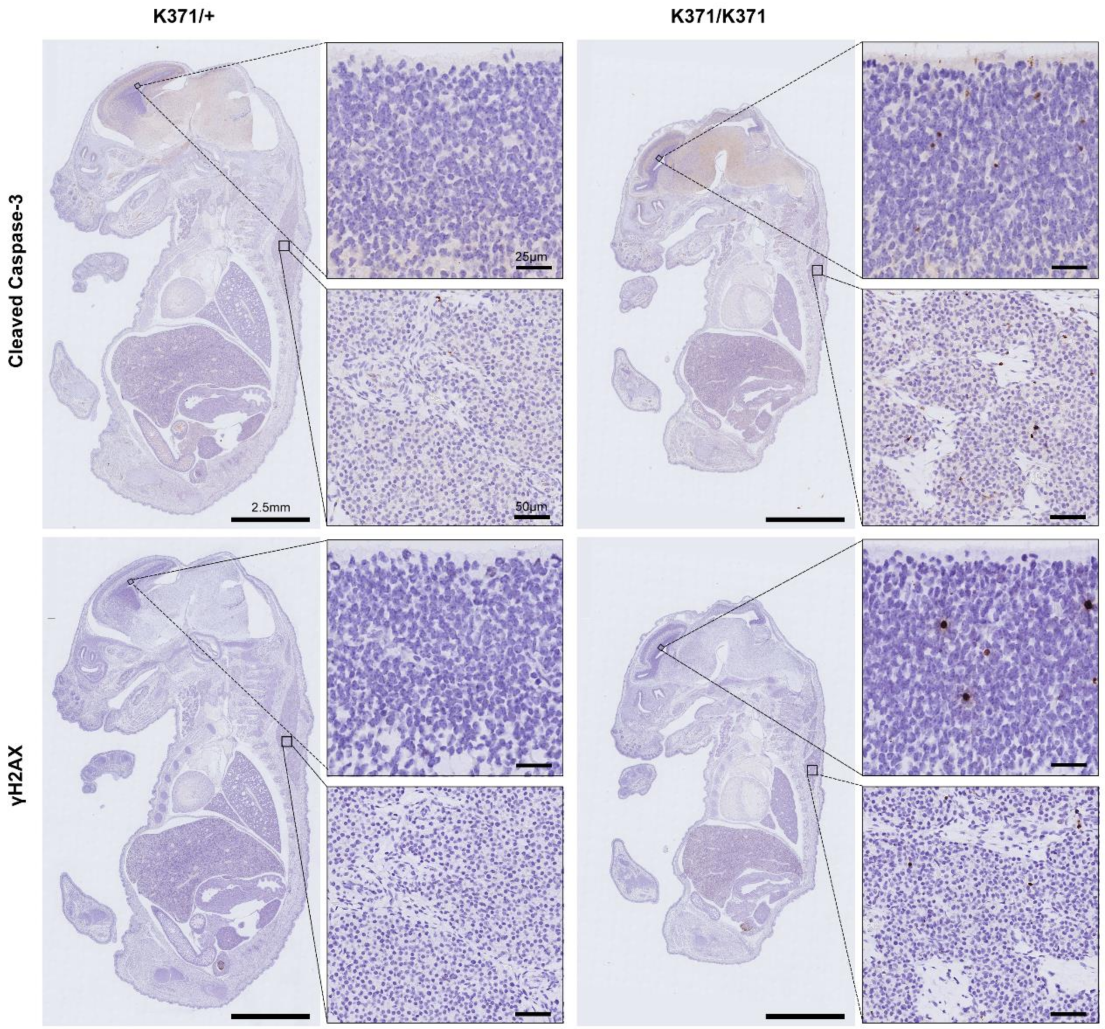

Figure S8: Smc5^{K371K371} embryos show increased DNA damage and apoptosis as compared to heterozygous embryos. IHC staining of Smc5$^{K371/+}$ and Smc5^{K371K371} embryos for γH2AX and cleaved caspase-3 at E15.5. Boxed regions in each embryo were magnified and showed details of brain and brown adipose precursors, respectively.

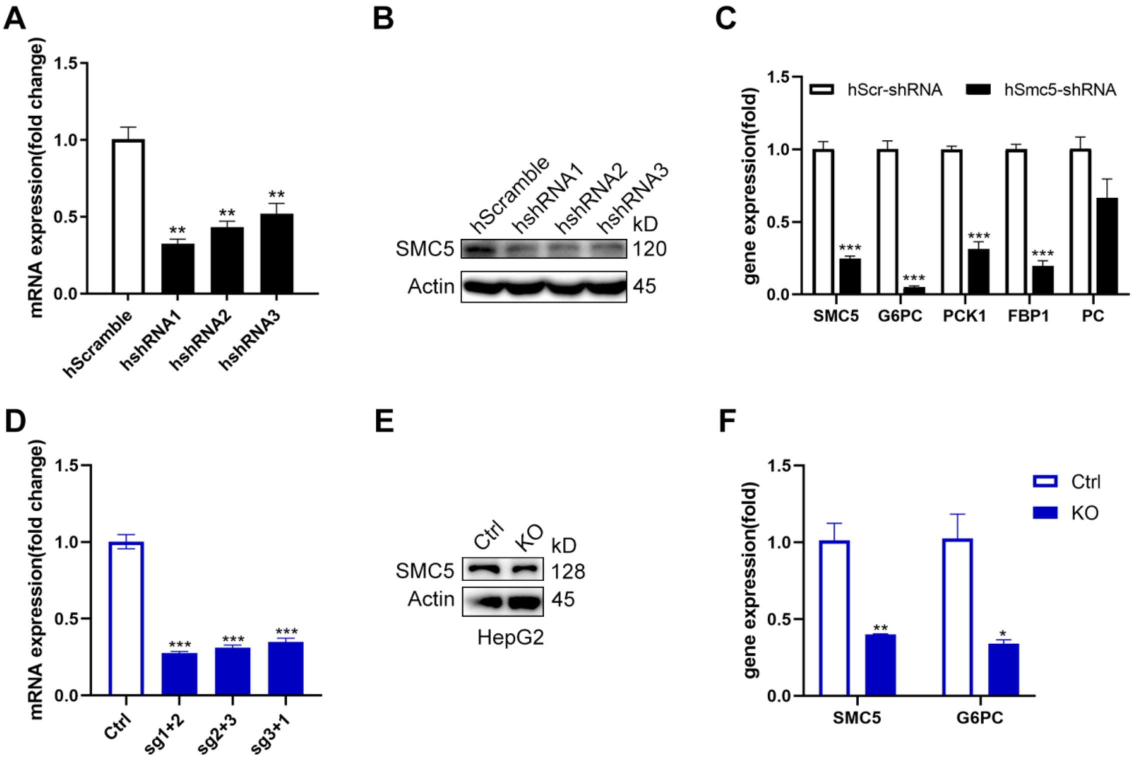

Figure S9: SMC5 deficiency impaired gluconeogenesis in HepG2 cells. (A-C) The HepG2 cells were infected with lentivirus shRNAs targeting SMC5 (hSmc5-shRNA) or scramble (hScr-shRNA) virus. qPCR (A) and western blot (B) confirmation of SMC5 knockdown efficiency. (C) qPCR of G6PC (encoding glucose 6-phosphatase), PCK1 (encoding phosphoenolpyruvate carboxykinase), FBP1 (encoding fructose 1–6 bisphosphatase), PC (encoding pyruvate carboxylase) expression. (D-F) The HepG2 cells were infected with lentivirus sgRNAs targeting SMC5 or control (Ctrl) virus. qPCR (D) and western blot (E) confirmation of SMC5 KO efficiency. (F) qPCR of G6PC expression in SMC5 KO cells. Actin was chosen as the internal reference gene. Values represent the mean ± s.e.m. *P < 0.05, **P < 0.01, ***P < 0.001 by Student's t.

Table S1: Clinical and hormonal characteristics of the patient.

Variable			Reference Range
Age (yr)	29	31	
BMI (kg/m^2)	21.1	20.4	
HbA1c (%)		9.4	4.7–6.3
HOMA-IR	41.90	24.54	
HOMA-IS	0.02	0.04	
IGF-1 (ng/ml)	282		117-329
IGFBP-3 (ug/ml)	4.30		3.5-7.6
ACTH (pg/ml)	31.1(8am);28.0(4pm)		0-46
Cortisol (ug/dl)	22.4(8am);5.47(4pm)		5-25(8am);<br>2.5-12.5 (4pm)
FSH (mIU/ml)	8.81	7.86	0.95-11.95
LH (mIU/ml)	6.89	5.51	0.57-12.07
Prolactin (ng/ml)	10.24	24.37	3.46-19.4
Estradiol (pg/ml)	49	63	11-44
Progesterone (ng/ml)	0.100	0.2	0-0.2
Testosterone			
Total (ng/ml)	6.25	6.96	1.42-9.23
Free (pg/ml)	10.96		8.69-54.69
17-OHP (ng/ml)	1.06		0.5-2.4
Androstenedione (ng/ml)	0.91	2.70	0.30-2.63*;0.7-3.6†
Dihydrotestosterone (pg/ml)	371.55		17.9-579
DHEA-S (ug/dl)	200.5	189	136.2-591.9*;80-560†

*Reference range tested at 29 years old; † Reference range tested at 31 years old. HOMA-IR index = Fasting insulin (mU/L)×Fasting glucose(mmol/L) /22.5**;** HOMA-IS index = 1/HOMA-IR[1]. Abbreviations: HbA1c, glycosylated hemoglobin; IGF-1, insulin growth factor-1; IGFBP-3, IGF binding protein-3; FSH, follicle-Stimulating Hormone; LH, luteinizing Hormone; 17-OHP, 17-α-hydroxy progesterone; DHEA-S, dehydroepiandrosterone sulfate.

Table S2: Growth hormone in stimulation test with arginine in the patient.

	Growth hormone (ng/ml)	
	29yr	31yr
Time (min)		
0	0.88*	0.045†
30	24.41	2.401
60	43.81	24.309
90	28.41	14.395
120	19.96	

*Reference range was 0-11.6 ng/ml tested at 29 years old.

† Reference range was 0.003-0.971 ng/ml tested at 31 years old.

Table S3: Details on the 11 candidate genes with homozygous mutations remaining after the filtering of WES data.

Gene	CHR:POS	Ref:Alt	AA Change	esp6500siv2_all	1000g2015aug_eas	ExAC_EAS	170normal	SIFT(score)	Polyphen2(HVAR score)
LYST	1:235866049	G:A	p.P3458S	NR	NR	0	NR	D(0.009)	B(0.062)
NID1	1:236144971	C:A	p.R1056L	NR	NR	NR	NR	D(0.005)	PrD(0.998)
PCDH9	13:67800537	G:A	p.P679L	NR	NR	0	NR	D(0.001)	PrD(0.951)
POLDIP2	17:26682828	G:A	p.THR79ILE	NR	NR	NR	NR	NA	PoD(0.606)
MYH1	17:10406128	T:A	p.D1013V	NR	NR	NR	NR	D(0)	PoD(0.846)
APBA3	19:3754321	G:A	p.H212Y	NR	NR	NR	NR	D(0.01)	B(0.25)
PNKP	19:50367485	T:C	p.Y196C	0.0005	NR	0	NR	D(0.002)	PoD(0.854)
CBLB	3:105412408	T:C	p.S613R	NR	NR	NR	NR	D(0.021)	B(0.069)
SIDT1	3:113286490	G:A	p.A150T	0.0002	NR	0	NR	D(0.004)	B(0.072)
SEZ6	17:27332838	G:T	p.A17D	NR	NR	NR	NR	D(0.006)	PoD(0.553)
SMC5	9:72912937	AGAG:A	p.R372del	NR	NR	NR	NR	NA	NA

Genomic information, variant frequency in the general population, and in silico prediction of functional impact for each variant. Abbreviations are as follows: NR=none reported, D=damaging, PrD=probably damaging, T=tolerated, PoD=possibly damaging.

Table S4: chromosomal aberration and micronucleus in lymphocytes from the patient.

**Cytogenetic analysis**	Patient	Control	Ref
The frequencies of chromosomal aberrations（%）	7	0	0-2
Metaphases	100	100	
Acentric fragments （%）	7	0	0-3
Dicentrics（%）	0	0	<1
Rings （%）	0	0	<1
Inversions （%）	0	0	<1
Translocations （%）	0	0	<1
Deletions （%）	0	0	<1
MN frequencies（‰）	2	0	0-6

Table S5. The target sequences for the shRNAs or sgRNAs in lentivirus vectors.

Gene	Species	Sequence 5'---3'
SMC5	HUMAN	hshRNA1: GCATTATGTGAAGGCGAAATA<br>hshRNA2: GCGAAACTTGTTACCGAATTA<br>hshRNA3: GAGGTGAAAGAAGTGTTTCTA
SMC5	HUMAN	hsg1: GGAGTTGACGTCTTCTTGCT<br>hsg2: ACCTCCGACGAAGGGTCTCT<br>hsg3: GGCCCGGACGACTGCAACAG
Smc5	Mouse	mshRNA1: CAGTAGTACTCAGCTGCATGAATTA<br>mshRNA2: TCATCACACCGAAGCTTCTACAGAA<br>mshRNA3: GATGACAGTGTTGTTTGTCTACAAT
NC	N/A	TTCTCCGAACGTGTCACGTAA

Table S6: Antisense morpholino oligonucleotides used in zebrafish.

Material	Sequence (5'- 3')
MO-Control	CCTCTTACCTCAGTTACAATTTATA
MO-smc5^{ATG}	TGGTTGATGTTCAGCACAGTCTATT
MO-tp53^{ATG}	TTGATTTTGCCGACCTCCTCTCCAC

Note: MO-Control: as a negative control for off-target MO toxicity; MO-smc5^{ATG}: to investigate if the smc5 down regulation can mimic dwarf phenotype in zebrafish embryos; MO-tp53^{ATG}: to rescue dwarf phenotype in smc5 knockout embryos.

Table S7: Primers used for qPCR and WISH.

Gene Symbol	Forward Primer (5'-3')	Reverse Primer (5'-3')
zebrafish (Primers for qPCR)		
smc5	AGTCCAGTATCGTGTGTGCCAT	CCTCTTTGACACCCTCTCTTCAC
chek2	AGCGTGCTCAATTGCACAGA	TCGTCGTCCACCCATGTG
tp53	GCAGCGATGAGGAGATCTTT	GGGCTCAGATGATTCACGAT
mdm2	CCGAGGCAGACTACTGGAAG	CGAAGGTTGTGTTGGGAGTT
ccng1	TCTCTCCTTGACTCGATTCTTTG	AATATTCAACCAGGCACTTAGCA
cdkn1a	AAACATCCCGAAAACACCAG	TACGCTGTACGAGACGATGC
ccnb2	CACAAACCAGTTCAGACAAAGAAGG	GCTGGACCTGGGCACTTTTG
ccna1	TATGAACCAACGCACCAGG	TCTGAAGGCAGCAGGAATGT
bbc3	CAGCACTCTCTCTGCACCAC	CCTCTGAGGAGCTCTGGTTG
casp8	CTCAAACGAACAGGCACTGA	ACAAAAGCACCCATTGAAGC
gadd45aa	TCTCATCCAGGCTTTCTGCT	GCAGAAGCGGTTCACTTTTC
cenpv	GGTGCGTTTTGAGGTGTGG	GCAGCAAGGTGAACTGGGA
h2afx	GTGTTCACCGTCTCCTTCGT	CCGGTCTTCTTGGGCAGGAG
pcna	GCTCGTCGGGCATTTCT	CAGAGGAGTGGCTTTGG
tdp1	GCTCCTCAATTGGCTTCCCT	ATGTTCCAGATCCAAGGCCG
msh5	ACATGCCGGACACTGTGGA	AGCATTGCTCTTGTTTGGCAC
actin	CGAGCAGGAGATGGGAACC	CAACGGAAACGCTCATTGC
zebrafish (Primers for WISH)		
insulin	ATGGCAGTGTGGCTTCAGGCT	GCAAAGTCAGCCACCTCAGTT
pdx1	CTTCACCTTCCCCAAACTTCAC	AAGTGTTGCCGTGCCGA
pax6b	GAGACTTGCGGCAAAAATTG	ATGAGTCCTGTGGAAGCGG
nuerod1	TCTTTTGTGCAGGCCTTGTG	TAAAGAGACGCGGCGTGTC
pcsk1	CATCTAATGGTTTCCGAAACTGG	AGCGGTTCAGCAGGTCTAGG
pcsk2	ACTGTAACTGTGATGGATACGCCTC	AGCATCCAGCACACCGTACC
cpe	GGAGGTATGCAGGACTTCAACT	GCGTCTCTGACATCATCTTCC
Mouse (Primers for qPCR)		
Smc5	TTGATTGAACGGAAGGAC	ATCATCTTGCGGGTGTTA
Fabp4	GGGGCCAGGCTTCTATTCC	GGAGCTGGGTTAGGTATGGG
Pparg	TCGCTGATGCACTGCCTATG	GAGAGGTCCACAGAGCTGATT
Cebpa	CAAGAACAGCAACGAGTACCG	GTCACTGGTCAACTCCAGCAC
Adipoq	GACAAGGCCGTTCTCTTCAC	CCAGATGGAGGAGCACAGAG
Trp53	GGCAGACTTTTCGCCACAG	CAGGCACAAACACGAACCTC
Cdkn1a	CGCTGTCTTGCACTCTGGT	CGTTTTCGGCCCTGAGATGTT
Mdm2	ATGAATCCTCCCCTTCCATC	CTGTCAGCTTTTTGCCATCA
Ccng1	ACAACTGACTCTCAGAAACTGC	CATTATCATGGGCCGACTCAAT
Pmaip1	GCAGAGCTACCACCTGAGTTC	CTTTTGCGACTTCCCAGGCA
Bcl2	ATGCCTTTGTGGAACTATATGGC	GGTATGCACCCAGAGTGATGC
Cdkn2a	CCCAACGCCCCGAACT	GCAGAAGAGCTGCTACGTGAA
Cdkn3	ATGAAGCCGCCCATTTCAATA	GGAAGAGCACATAAACCGAGAA
34b4	CTGAAGTGCTCGACATCACAGAG	GCTTGTACCCATTGATGATGGAG
HUMAN (Primers for qPCR)		
G6PC	TCATCTTGGTGTCCGTGATCG	TTTATCAGGGGCACGGAAGTG

PCK1	GAAAAAACCTGGGGCACAT	TTGCTTCAAGGCAAGGATCTCT
FBP1	CGCGCACCTCTATGGCATT	TTCTTCTGACACGAGAACACAC
PC	ACAGAGGTGAGATTGCCATCC	CACTGCATCTACGTTGTTCTCC
ACTIN	CATGTACGTTGCTATCCAGGC	CTCCTTAATGTCACGCACGGAT

Table S8: Sequence of sgRNA and ssODN.

Material	Sequence (5’- 3’)
sgRNA	TGTTACTTATTCTCTTCTGCCGG
ssODN	TTAGATTAAGGAACTTCAGCAGGCATTAACAGTCAAGCAGAAT<br>GAAGAGCTGGACCGGCAG(AAG)AGAATAAGTAACACCCGCAA<br>GATGATAGAGGATCTGCAGAGTGAGCTCAAGACTGCGGA

Note: The bases underlined are PAM sequence. The gRNA is antisense to the mouse Smc5 sequence; The brackets indicate mutant nucleotides that delete K371, and concurrently destroy the corresponding gRNA cut site.

Table S9: RNA-seq analysis of WT and smc5 KO 3 dpf larvae (has been submitted independently as an excel file). Differential expressed genes (DEGs) with FDR < 0.05 and |log2FC| ≥0.58.

Table S10 (Related to Figure 4F): The number of embryos of specific genotypes observed at 5dpf from smc5$^{+/-}$; p53$^{+/-}$ zebrafish intercrosses.

Genotype		Number	Expected	Body length
smc5	p53	(Total=140)	number	(mean±SD; μm)
+/+	+/+	8	9	3819±103
+/+	+/-	19	17	3783±116
+/+	-/-	6	9	3677±112
+/-	+/+	17	17	3814±93
+/-	+/-	38	34	3826±104
+/-	-/-	23	17	3771±132
-/-	+/+	6	9	3677±93
-/-	+/-	14	17	3843±89
-/-	-/-	9	9	3824±93

Supplemental References

1. Matthews DR, Hosker JP, Rudenski AS, Naylor BA, Treacher DF, Turner RC. Homeostasis model assessment: insulin resistance and beta-cell function from fasting plasma glucose and insulin concentrations in man. *Diabetologia.* 1985;28(7):412-419.
